# Rpb7 represses transcription-coupled nucleotide excision repair

**DOI:** 10.1016/j.jbc.2023.104969

**Published:** 2023-06-27

**Authors:** Wenzhi Gong, Shisheng Li

**Affiliations:** Department of Comparative Biomedical Sciences, School of Veterinary Medicine, Louisiana State University, Baton Rouge, Louisiana, USA

**Keywords:** Rpb7, Rpb4, transcription couped repair, Spt4, Spt5, Rad26

## Abstract

Transcription-coupled repair (TCR) is a subpathway of nucleotide excision repair (NER) that is regulated by multiple facilitators, such as Rad26, and repressors, such as Rpb4 and Spt4/Spt5. How these factors interplay with each other and with core RNA polymerase II (RNAPII) remains largely unknown. In this study, we identified Rpb7, an essential RNAPII subunit, as another TCR repressor and characterized its repression of TCR in the *AGP2*, *RPB2*, and *YEF3* genes, which are transcribed at low, moderate, and high rates, respectively. The Rpb7 region that interacts with the KOW3 domain of Spt5 represses TCR largely through the same common mechanism as Spt4/Spt5, as mutations in this region mildly enhance the derepression of TCR by *spt4Δ* only in the *YEF3* gene but not in the *AGP2* or *RPB2* gene. The Rpb7 regions that interact with Rpb4 and/or the core RNAPII repress TCR largely independently of Spt4/Spt5, as mutations in these regions synergistically enhance the derepression of TCR by *spt4Δ* in all the genes analyzed. The Rpb7 regions that interact with Rpb4 and/or the core RNAPII may also play positive roles in other (non-NER) DNA damage repair and/or tolerance mechanisms, as mutations in these regions can cause UV sensitivity that cannot be attributed to derepression of TCR. Our study reveals a novel function of Rpb7 in TCR regulation and suggests that this RNAPII subunit may have broader roles in DNA damage response beyond its known function in transcription.

Nucleotide excision repair (NER) is a highly conserved DNA damage repair mechanism that removes bulky and/or helix-distorting DNA lesions, such as ultraviolet (UV) induced cyclobutane pyrimidine dimers (CPDs) (1, 2, 3, 4). NER occurs through a process that is remarkably similar in organisms from bacteria and yeast to the more complex plants and mammals. Two subpathways of NER exist, with the first being global genomic repair (GGR), which removes lesions throughout the genome, including the nontranscribed strand (NTS) of actively transcribed genes. In the yeast *Saccharomyces cerevisiae*, Rad7, Rad16, and Elc1, which form a complex, play important roles in the damage recognition step during GGR (5, 6, 7). Deletion of either Rad7 or Rad16 abolishes GGR (7), whereas deletion of Elc1 greatly compromises GGR (5).

Transcription-coupled repair (TCR) is another subpathway of NER that is dedicated to the rapid removal of lesions in the transcribed strand (TS) of actively transcribed genes (8, 9, 10, 11, 12, 13). TCR is not only restricted to the “canonical” TS” but can also accompany cryptic antisense transcription in certain genes where canonical transcription is largely repressed (14) or where cryptic transcription is derepressed due to certain mutations that affect transcription (15). In eukaryotic cells, TCR is triggered by the stalling of RNA polymerase II (RNAPII), a 12-subunit (Rpb1-12) complex with Rpb4/Rpb7 forming a subcomplex that interacts with the 10-subunit core RNAPII mainly through a “tip” of Rpb7 (16, 17). In yeast, TCR has been shown to be facilitated by multiple proteins, including Rad26, a DNA-dependent ATPase (18, 19), Rpb9, a nonessential subunit of RNAPII (20), Sen1, an ATPase-helicase (21), and Elf1, a transcription elongation factor (22, 23). TCR in yeast has also been demonstrated to be repressed by multiple proteins, including Rpb4, another nonessential subunit of RNAPII (20), the Spt4/Spt5 complex, which promotes transcription elongation (24, 25), and the RNAPII–associated factor complex (PAFc) (26).

Rad26 (27) and its human homolog CSB (28) contact the upstream DNA of the RNAPII elongation complex and displace Spt4/Spt5 (28, 29). The remarkable structural similarity between yeast and humans suggests that the mechanisms underlying TCR in both systems are fundamentally similar. As a result, it is likely that both yeast Rad26 and human CSB play a role in facilitating TCR by antagonizing TCR repressors. In addition to displacing Spt4/Spt5, human CSB has been shown to directly recruit NER factors to the RNAPII complex, as it interacts with CSA (30, 31), which in turn interacts with the NER factors UVSSA and DDB1/DDB2 (28, 32, 33, 34). How different TCR repressors function cooperatively or individually to oppose the function of Rad26 remains largely unknown.

As exemplified by the opposing roles of Rpb4 and Rpb9, which repress and facilitate TCR, respectively (20), different RNAPII subunits may regulate TCR very differently, presumably due to their different locations on RNAPII and different interactions with other transcription elongation and/or NER factors. Although it forms a subcomplex with Rpb4, how Rpb7 functions in TCR in the cell remains difficult to address, largely due to its essentiality for cell survival. Through unbiased screening of random mutations and site-directed mutagenesis, we identified multiple Rpb7 mutants that derepress TCR. Our results indicate that Rpb7 may repress TCR through interactions with not only Rpb4 but also the KOW3 domain of Spt5 and core RNAPII. The region of Rpb7 that interacts with the KOW3 domain of Spt5 may repress TCR largely through the same common mechanism as Spt4/Spt5. On the other hand, regions of Rpb7 that interact with Rpb4 and/or the core RNAPII may repress TCR largely independent of Spt4/Spt5. Our results also suggest that Rpb7 plays a positive role in other (non-NER) DNA damage repair and/or tolerance mechanisms.

## Results

### Screening of random Rpb7 mutants identified G149 as an important residue for the repression of TCR

**Figure 1:**
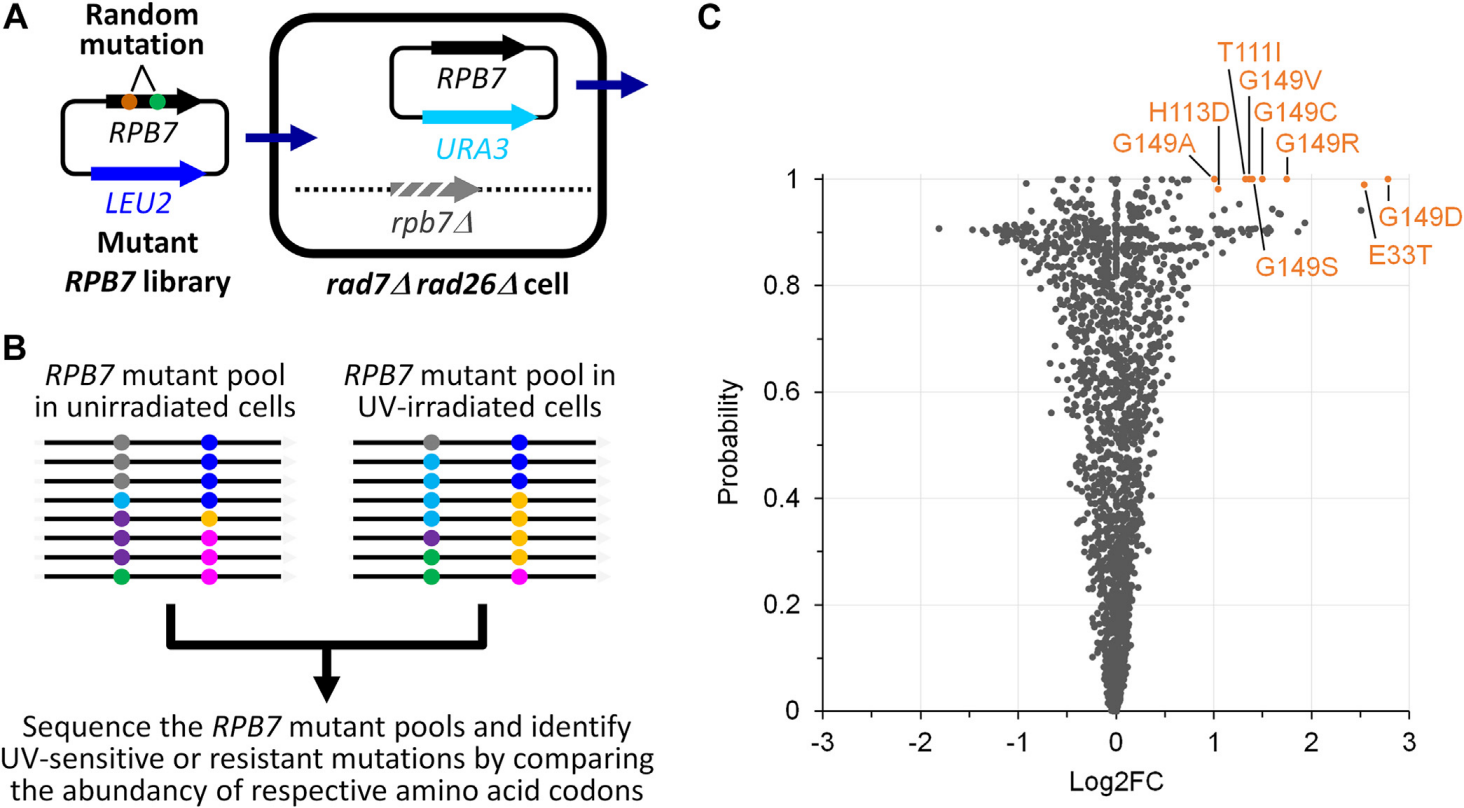
**Screening of random Rpb7 mutants.***A*, shuffling of a *LEU2* plasmid library bearing *RPB7* gene molecules randomly mutagenized at codons 2 to 171 into *rad7Δ rad26Δ* cells whose genomic *RPB7* had been deleted and complemented with the *URA3* plasmid bearing the wild type *RPB7* gene. *B*, assessments of UV sensitivities of Rpb7 mutants by next-generation DNA sequencing. *Black lines* indicate the *LEU*-plasmid-born *RPB7* gene molecules with random mutations denoted as *dots* of different colors. *C*, plot showing the Log2FC changes and probabilities of Rpb7 mutants in UV-irradiated *rad7Δ rad26Δ* cells compared to unirradiated counterparts. The Rpb7 mutants exhibiting Log2FC >1 and probability >0.98 are indicated.

The highly efficient GGR in yeast cells can mask the effects of a TCR facilitator or repressor on UV sensitivity and the repair event (35). By eliminating or mutating a protein in GGR-defective *rad7Δ* (or *rad16Δ*) cells, the role of the protein in regulating TCR may be unambiguously assessed. To investigate the role of Rpb7, which is essential for cell viability, in TCR, we started with screening UV-sensitive or resistant random Rpb7 mutants in *rad7Δ rad26Δ* cells (Fig. 1, *A* and *B*). If Rpb7 facilitates or represses TCR, its viable mutants may make *rad7Δ rad26Δ* cells UV sensitive or resistant, respectively. Our screening covered 3344 (98.4%) of all 3400 (20 × 170) possible *RPB7* codons at positions 2 to 171, although the counts for some of the Rpb7 mutant codons were low (Table S1). The Rpb7 G149D mutant (rpb7G149D) was the most enriched one in the UV-irradiated cells (θ = 3.42, probability = 1 and log2 fold change (log2FC) = 2.78) (Fig. 1*C* and Table S2). All other G149 mutants covered in the screening, including G149A, G149C, G149E, G149R, G149S, G149V and G149Y, were also enriched to some extent in the UV-irradiated cells (probability > 0.9 and log2FC > 1) (Fig. 1*C* and Table S2). These results suggest that Rpb7 G149 may be important for repressing TCR; its mutation may derepress TCR and make *rad7Δ rad26Δ* cells UV resistant. The positive log2FC, θ, and/or probability values of mutants at other Rpb7 codons were not as large as those at G149. None of the Rpb7 mutants whose log2FC < −1 had a θ value < 0.81 or a probability value >0.92 (Table S2).

**Figure 2:**
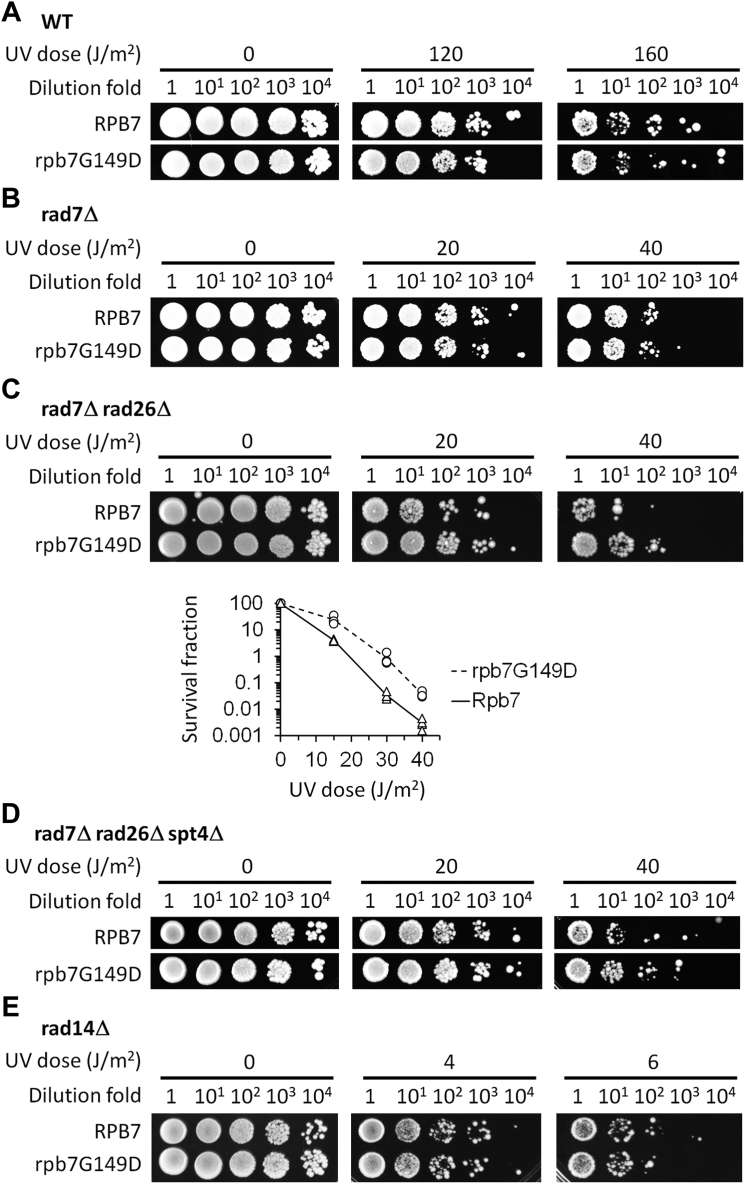
**Effects of rpb7G149D on UV sensitivity.***A–E*, images of spotted yeast cells of indicated genotypes following irradiation with the indicated UV doses. The plot shown at the *bottom* of (*C*) displays the survival fraction of *rad7Δ rad26Δ* cells expressing either wild-type Rpb7 or the rpb7G149D mutant. The values at indicated UV doses were obtained from four independent experiments, and the lines shown represent the mean values of these experiments. In each panel, RPB7 (wild type) and rpb7G149D mutant cells were on the same plate of each of the indicated UV doses.

We wanted to confirm the effect of rpb7G149D on TCR by creating yeast strains with the specific mutation. Indeed, rpb7G149D increased the UV resistance of *rad7Δ rad26Δ* cells ∼10-fold (Fig. 2*C*). rpb7G149D also increased the UV resistance of *rad7Δ rad26Δ spt4Δ* cells ∼5 fold (Fig. 2*D*) but did not affect the UV resistance of otherwise wild type, *rad7Δ* or *rad14Δ* (completely NER-defective) cells (Fig. 2, *A*, *B*, and *E*). These results support the idea that rpb7G149D derepresses TCR in the absence of Rad26 but may not significantly affect GGR or any non-NER DNA repair mechanisms.

We then directly analyzed the effect of rpb7G149D on the repair of UV-induced CPDs. Different TCR facilitators may contribute differently to the repair event in genes with different transcription levels (8, 35). For example, Rad26 plays a more important role in TCR in slowly transcribed genes than in highly transcribed ones (8, 35). How TCR repressors function in genes with different transcription levels is essentially undocumented. To gain insights into how a TCR repressor (or facilitator) regulates TCR, it is ideal to analyze its effects on the repair event in genes with different transcription levels. Although next-generation sequencing technology has been available, genome-wide repair analyses in a large number of mutant cells at multiple repair time points can still be prohibitively exhausting. We therefore chose to analyze CPD repair in the *AGP2*, *RPB2,* and *YEF3* genes, which are transcribed at low, moderate, and high speeds, respectively (36). Yeast cells were cultured to late log phase and UV-irradiated. At different times of repair incubation, genomic DNA was isolated, restricted to release the gene fragments of interest, and incised at CPD sites. The *AGP2*, *RPB2,* and *YEF3* gene fragments were fished out using biotinylated oligonucleotides and streptavidin magnetic beads, ligated with a common adapter, and sequenced. The sequencing reads corresponding to counts of CPDs at individual sites were counted. To “visualize” the CPD levels at individual sites, pseudo images were prepared by converting the counts of sequencing reads to band intensities using R.

**Figure 3:**
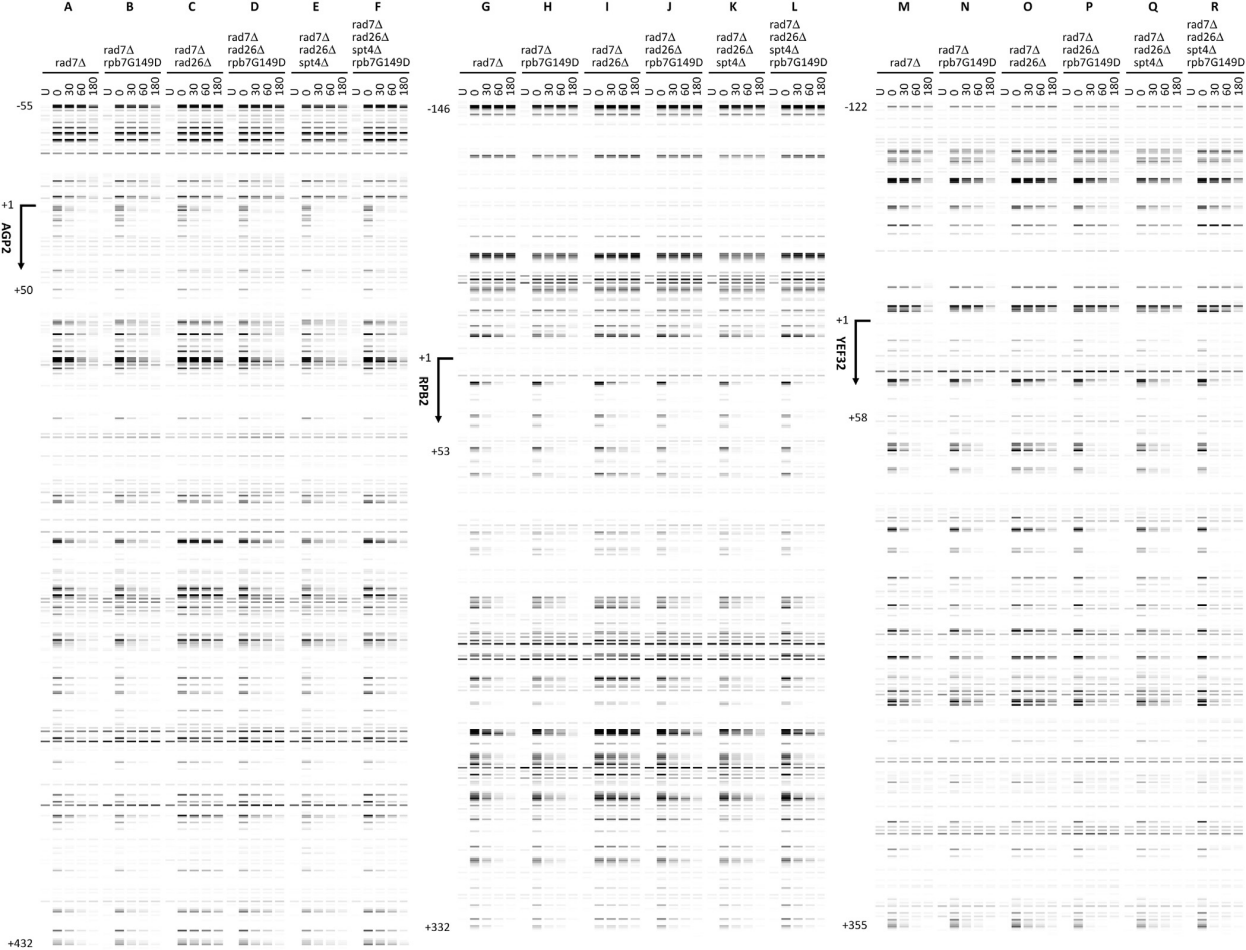
**Pseudo images showing effects of rpb7G149D on TCR.***A*–*R*, bands reflecting CPD levels along the TS of *AGP2*, *RPB2,* and *YEF3* genes in cells of indicated genotypes at the indicated repair times (min). ‘*U*’, sample unirradiated with UV. Numbers indicating the nucleotide positions of the genes are relative to the major TSS (+1).

**Figure 4:**
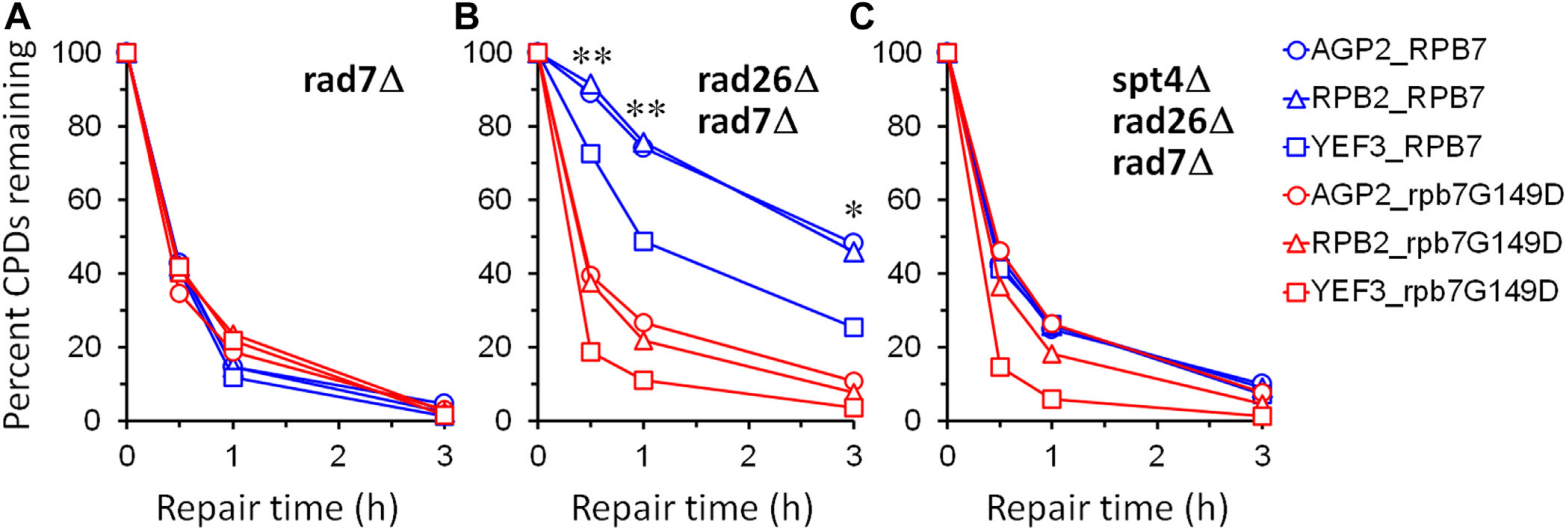
**Plots showing effects of rpb7G149D on TCR.***A*–*C*, means of percent CPDs remaining at all CPD sites 50 nucleotides downstream of the major TSS in the TS of *AGP2*, *RPB2*, and *YEF3* genes in cells with the wild-type or mutant Rpb7. ∗∗ and ∗ indicate *p* values < 0.01 and 0.05, respectively, at the respective repair time points (paired Student’s *t* test). The values for the wild type and mutant Rpb7 were paired with respect to the *AGP2*, *RPB2*, and *YEF3* genes for the Student’s *t* test.

In *rad7Δ* cells, rapid repair of CPDs can be seen immediately downstream of the major transcription start site (TSS) in the TS of these genes (Fig. 3, *A*, *G*, and *M*), indicating rapid TCR. TCR was slow in *rad7Δ rad26Δ* cells (Fig. 3, *C*, *I*, and O), especially in the region over 50 nucleotides downstream of the TSS, where the RNAPII switches to transcription elongation mode and TCR is repressed in the absence of Rad26 (35). TCR in the *YEF3* gene was not as slow as that in the *AGP2* or *RPB2* genes in *rad7Δ rad26Δ* cells (Fig. 3, compare O with C and I; Fig. 4*B*, compare blue symbols), agreeing with previous findings that TCR is less dependent on Rad26 in rapidly transcribed genes (20, 37). TCR was restored in *rad7Δ rad26Δ spt4Δ* cells (Figs. 3, *E*, *K*, and *Q* and 4, compare blue symbols among A, B, and C), agreeing with previous findings that *spt4Δ* derepresses TCR in *rad26Δ* cells (24, 38). rpb7G149D completely restored TCR in *rad7Δ rad26Δ* cells (Fig. 3, compare D with A and C, J with G and I, and P with M and O; Fig. 4, compare A and B) but did not substantially affect TCR in *rad7Δ* cells (Fig. 3, compare A and B, G and H, and M and N; Fig. 4*A*). These results indicate that rpb7G149D derepresses TCR in the absence of Rad26 but does not significantly affect TCR if Rad26 is present. In *rad7Δ rad26Δ spt4Δ* cells, rpb7G149D significantly enhanced TCR in the *YEF3* gene but not in the *AGP2* or *RPB2* gene (Fig. 3, compare E and F, K and L, and Q and R; Fig. 4*C*, compare blue and red symbols). This indicates that rpb7G149D enhances the derepression of TCR by *spt4Δ* in the rapidly transcribed gene, which is in line with our observation that rpb7G149D moderately increased the UV resistance of *rad7Δ rad26Δ spt4Δ* cells (Fig. 2*D*).

To determine the effect of rpb7G149D on GGR, we analyzed the repair of CPDs in the NTS of the *AGP2*, *RPB2,* and *YEF3* genes. In agreement with previous reports (*e.g.*, (39, 40)), GGR was affected by the positioning of nucleosomes, being slow and fast in the nucleosome core and linker regions, respectively (Figs. S1 and S2). rpb7G149D did not substantially affect GGR (Figs. S1 and S2).

### Rpb7 G149 may closely interact with the KOW3 domain of Spt5

**Figure 5:**
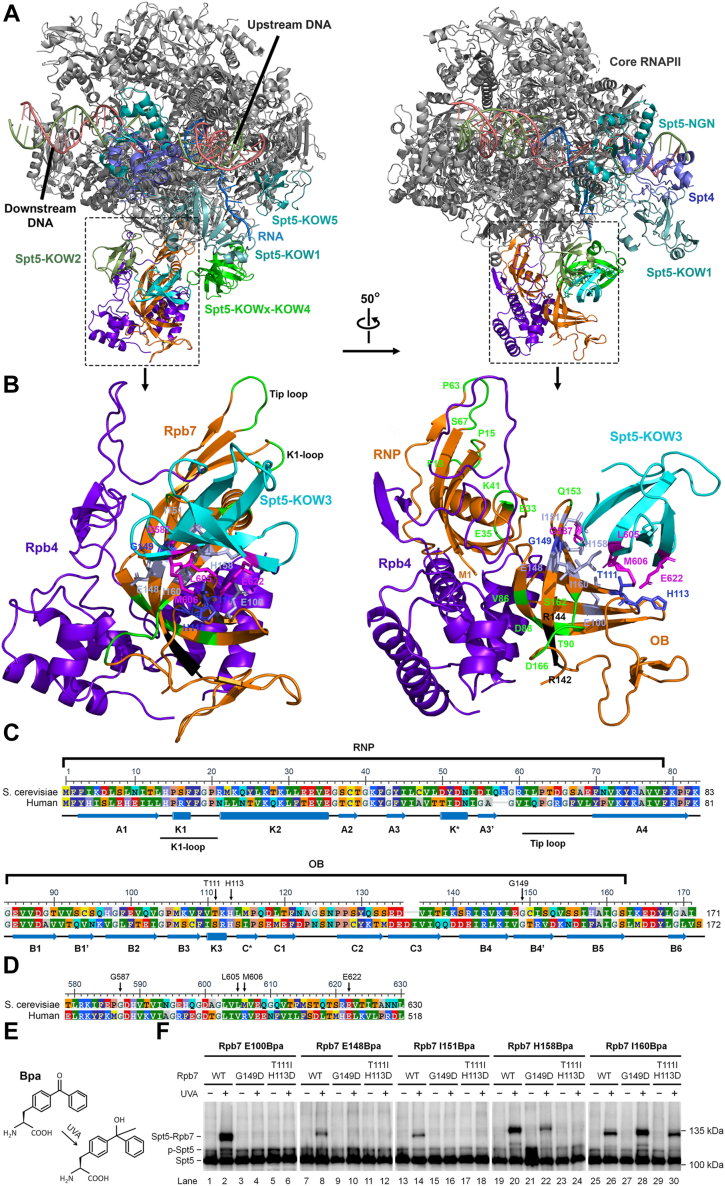
**Rpb7 residues implicated in repression of TCR.***A*, a model of yeast RNAPII complexed with Spt4 and Spt5 based on alignments of the substructures of yeast Spt4/Spt5 and RNAPII to the PDB file 6TED (structure of human transcription complex). The yeast substructures for the core RNAPII, Spt4, and the NGN and KOW5 of Spt5 are from 7NKY, those for Rpb4 and Rpb7 are from 1Y1W, that for Spt5 KOW1-linker is from 4YTK, that for Spt5 KOW2-3 is from 4YTL, and that for Spt5 KOWx-4 is modeled based 5OHO using Phyre2 (61). *B*, structure of Rpb4/Rpb7 complexed with Spt5 KOW3. Rpb7 residues G149, T111 and H113, which interact with Spt5 KOW3 and mutations of which (rpb7G149D and rpb7T111I-H113D) enhance TCR in *rad7Δ rad26Δ* cells (see Figure 3, Figure 4, Figure 7 and 8), are shown in *blue*. Rpb7 regions that interact with the core RNAPII, and/or Rpb4 and mutations of which (see Table 1) enhance TCR in *rad7Δ rad26Δ* cells (see Figs. 9 and S8–S10) are shown in *green*. The region of Rpb7 residues 142 to 144, which loosely interacts with Rpb4 and mutation of which (rpb7OB-6, Table 1) enhances TCR in *rad7Δ rad26Δ spt4Δ* cells but not in *rad7Δ rad26Δ* cells (compare Fig. 10*D* with Fig. 9*H*), are shown in *black*. *C*, alignment of the yeast and human Rpb7 protein sequences. *Cylinders* and *arrows* indicate regions of α-helices and β-strands, respectively. *D*, alignment of the yeast and human Spt5 KOW3 sequences. *E*, structure of Bpa. *F*, Western blots showing the effects of rpb7G149D and rpb7T111I-H113D mutations on the crosslinking of Bpa-substituted Rpb7 with 3xFLAG-tagged Spt5. The blots were probed with anti-FLAG antibody, and Bpa-substituted Rpb7 residues are indicated at the *top* of the blots. “Spt5-Rpb7” indicates Bpa-substituted Rpb7 crosslinked to Spt5, while “p-Spt5” stands for phosphorylated Spt5, which disappears upon phosphatase treatment or deletion of Spt5 CTR (C-terminal repeat) or the BUR2 gene (24).

The Rpb7 structure is composed of the N-terminal RNP (ribonucleoprotein-like) and the C-terminal OB (oligo-binding) domains (16, 41) (Fig. 5, *A*–*C*). The Rpb4/Rpb7 subcomplex interacts with the 10-subunit core RNAPII primarily through the Rpb7 tip consisting of the K1-loop and tip loop in the RNP domain (Fig. 5, *A*–*C*). The Rpb7 G149 is on the OB surface opposite the side of Rpb4 binding (Fig. 5*B*). Eukaryotic Spt5 proteins are composed of multiple conserved domains, including the NGN, KOWs (KOW1-5), and the C-terminal repeat (CTR) domain (42). Structures of human transcription complexes containing Spt4/Spt5 showed that Rpb7 G150, which corresponds to the yeast Rpb7 G149 (Fig. 5*C*), closely interacts with Spt5 G475, which corresponds to the yeast Spt5 G587 (Fig. 5*D*) and is located on the KOW3 domain of Spt5 (43). The yeast Spt5 NGN and KOW5 domains have been shown to interact with the core RNAPII “clamp”and “wall” domains, respectively (44). How the other domains of the yeast Spt5 interact with the yeast core RNAPII, Rpb4, or Rpb7 remains unresolved on the structural level. We aligned the yeast Rpb4, Rpb7, core RNAPII, Spt4, and Spt5 to the human counterparts (PDB 6ted) to generate a yeast structural model, which indicates that the yeast Rpb7 G149 may closely interact with G587 on the surface of the yeast Spt5 KOW3 (Fig. 5*B*). In support of this model, our previous studies with the photoreactive unnatural amino acid Bpa (p-benzoyl-L-phenylalanine) (Fig. 5*E*) substituting Rpb7 E100, E148, I151, H158 and I160 (E100Bpa, E148Bpa, I151Bpa, H158Bpa, or I160Bpa), which are adjacent to G149 on the Rpb7 surface, indicated that these Rpb7 residues closely interact with Spt5 (45). A Bpa-substituted protein can crosslink to its interacting proteins within a short distance (∼3 Å) upon UVA (350–365 nm) irradiation (46). To investigate if rpb7G149D affects these interactions, we generated yeast strains expressing Rpb7 with the G149D mutation and its Bpa-substituted counterparts (E100Bpa, E148Bpa, I151Bpa, H158Bpa, or I160Bpa). We found that rpb7G149D abolished crosslinking of Rpb7 E100Bpa, E148Bpa, or I151Bpa with Spt5, as evidenced by the lack of shifted Spt5 band on Western blots (Fig. 5*F*, compare lanes 3–4 with 1–2, 9–10 with 7–8, and 15–16 with 13–14). Moreover, the crosslinking of Rpb7 H158Bpa with Spt5 was dramatically reduced in the rpb7G149D mutant (Fig. 5*F*, compare lanes 21–22 with 19–20). Interestingly, the crosslinking of Rpb7 I160Bpa with Spt5 was increased in the rpb7G149D mutant (Fig. 5*F*, compare lanes 27–28 with 25–26). These results suggest that rpb7G149D dramatically affects the interaction between the Rpb7 OB surface with Spt5 KOW3.

### Rpb7 T111 and H113 are also important for repressing TCR

We wondered if other Rpb7 residues that may closely interact with Spt5 KOW3 also play a role in repressing TCR. Rpb7 T111 and H113 are on the OB surface area that may closely interact with the Spt5 KOW3 surface area formed by L605, M606, and E622 (Fig. 5*B*). Our screening of Rpb7 random mutations indicated that the Rpb7 T111I and H113D mutations confer *rad7Δ rad26Δ* cells mild UV resistance (log2FC > 1, θ > 1.4, probabilities > 0.98) (Fig. 1*C* and Table S2). We reasoned that simultaneous mutations of Rpb7 T111 and H113 (rpb7T111I-H113D) may synergistically enhance the effects of the individual mutations on the UV resistance. We found that rpb7T111I-H113D abolished crosslinking of Rpb7 E100Bpa, E148Bpa, I151Bpa, or H158Bpa with Spt5 (Fig. 5*F*, compare lanes 5–6 with 1–2, 11–12 with 7–8, 17–18 with 13–14, and 23–24 with 19–20). However, it did not affect the crosslinking of Rpb7 I160Bpa with Spt5 (Fig. 5*F*, lanes 29–30 compared to 25–26). These results suggest that rpb7T111I-H113D also dramatically affects the interaction between the Rpb7 OB surface and Spt5 KOW3.

**Figure 6:**
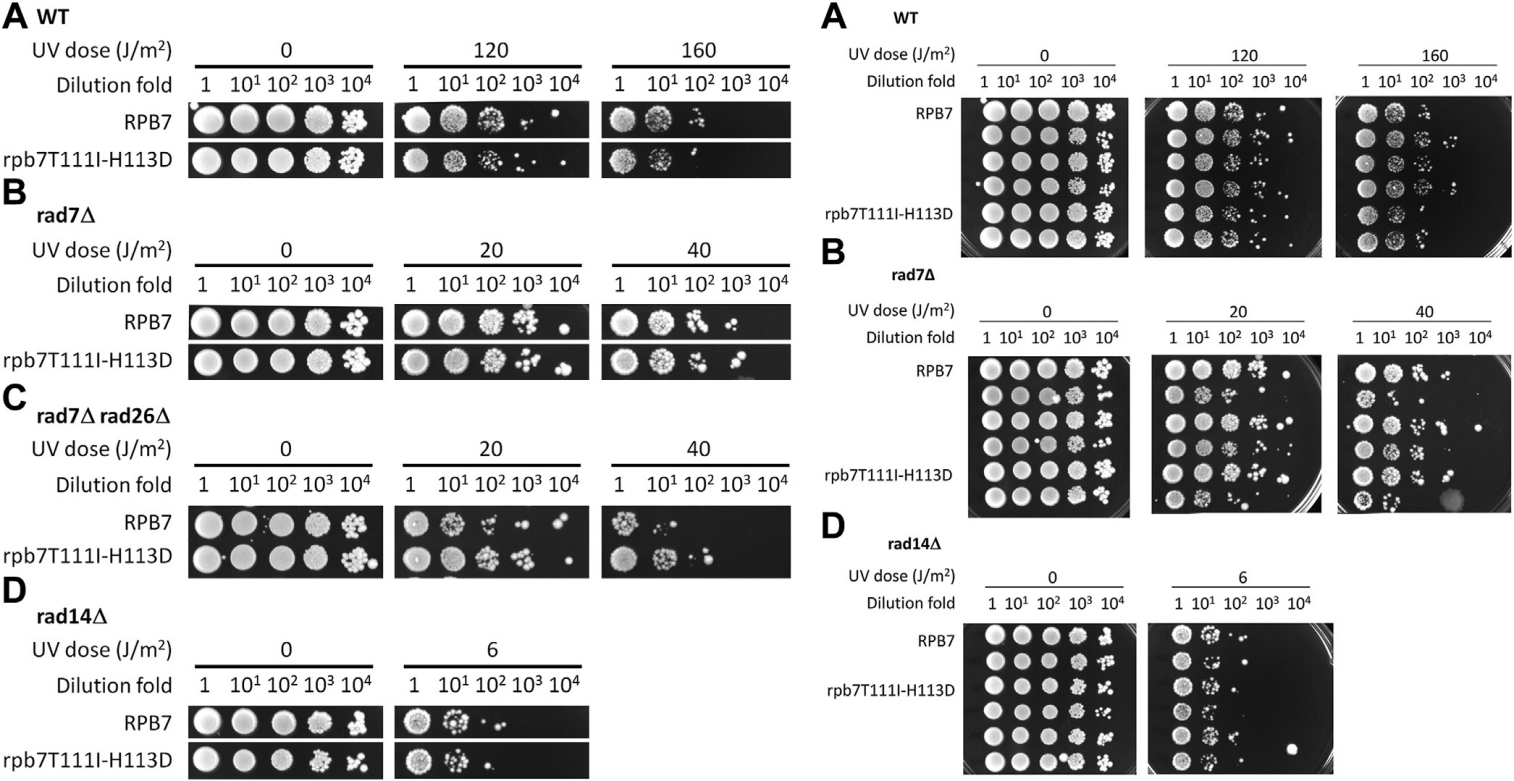
**Effects of rpb7T111I-H113D on UV sensitivity.***A*–*D*, images of spotted yeast cells of indicated genotypes following irradiation with the indicated UV doses. In each panel, RPB7 (wild type) and rpb7T111I-H113D mutant cells were on the same plate of each of the indicated UV doses.

**Figure 7:**
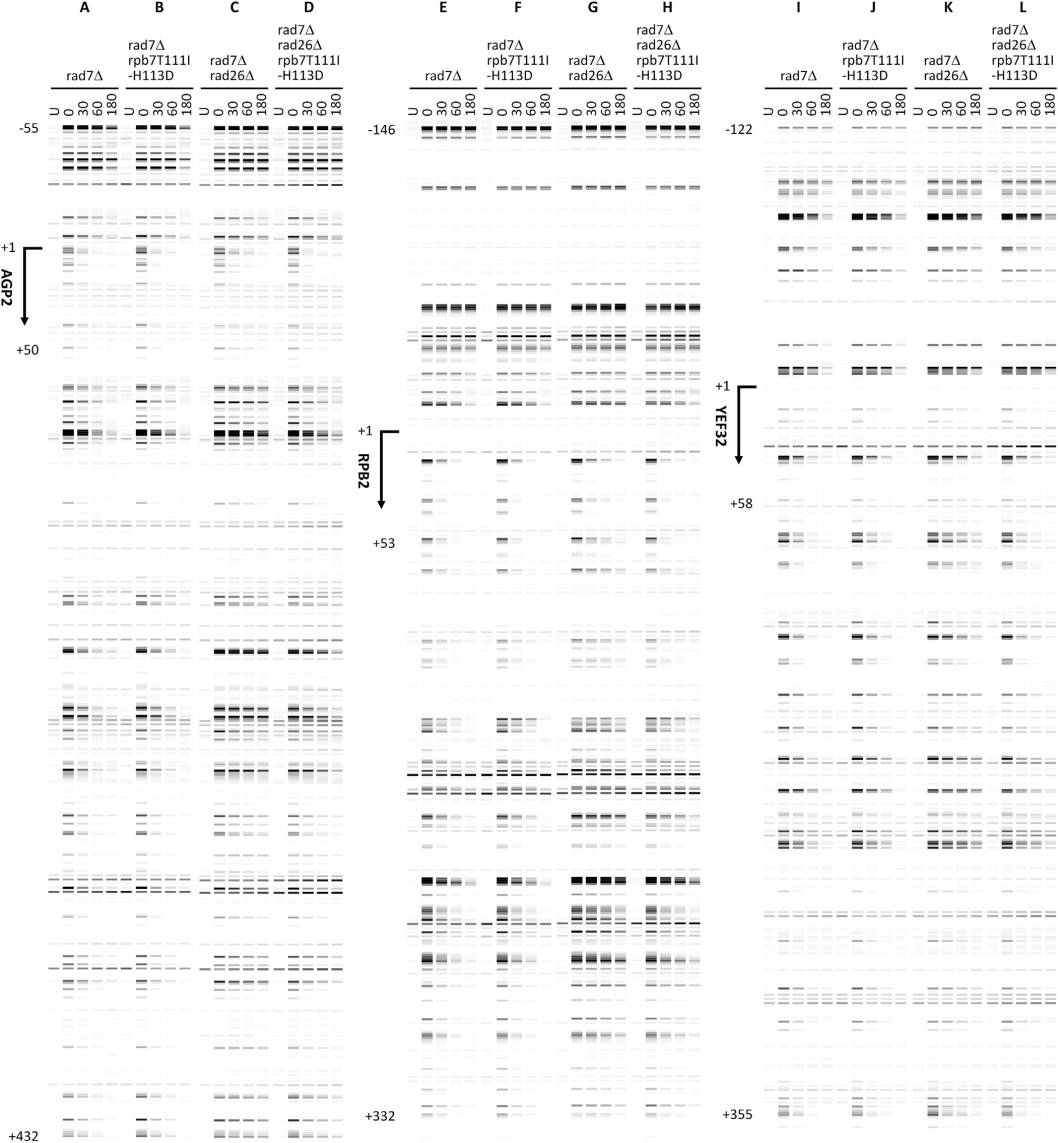
**Pseudo images showing effects of rpb7T111I-H113D on TCR.***A*–*L*, bands reflecting CPD levels along the TS of *AGP2*, *RPB2* and *YEF3* genes in cells of indicated genotypes at the indicated repair times (min). ‘*U*’, sample unirradiated with UV. Numbers indicating the nucleotide positions of the genes are relative to the major TSS (+1). Note that panels *A*, *C*, *E*, *G*, *I*, and *K*, which serve as wild-type Rpb7 controls for comparison with Rpb7 mutants, are the same as Figure 3, *A*, *C*, *G*, *I*, *M*, and *O*, respectively.

**Figure 8:**
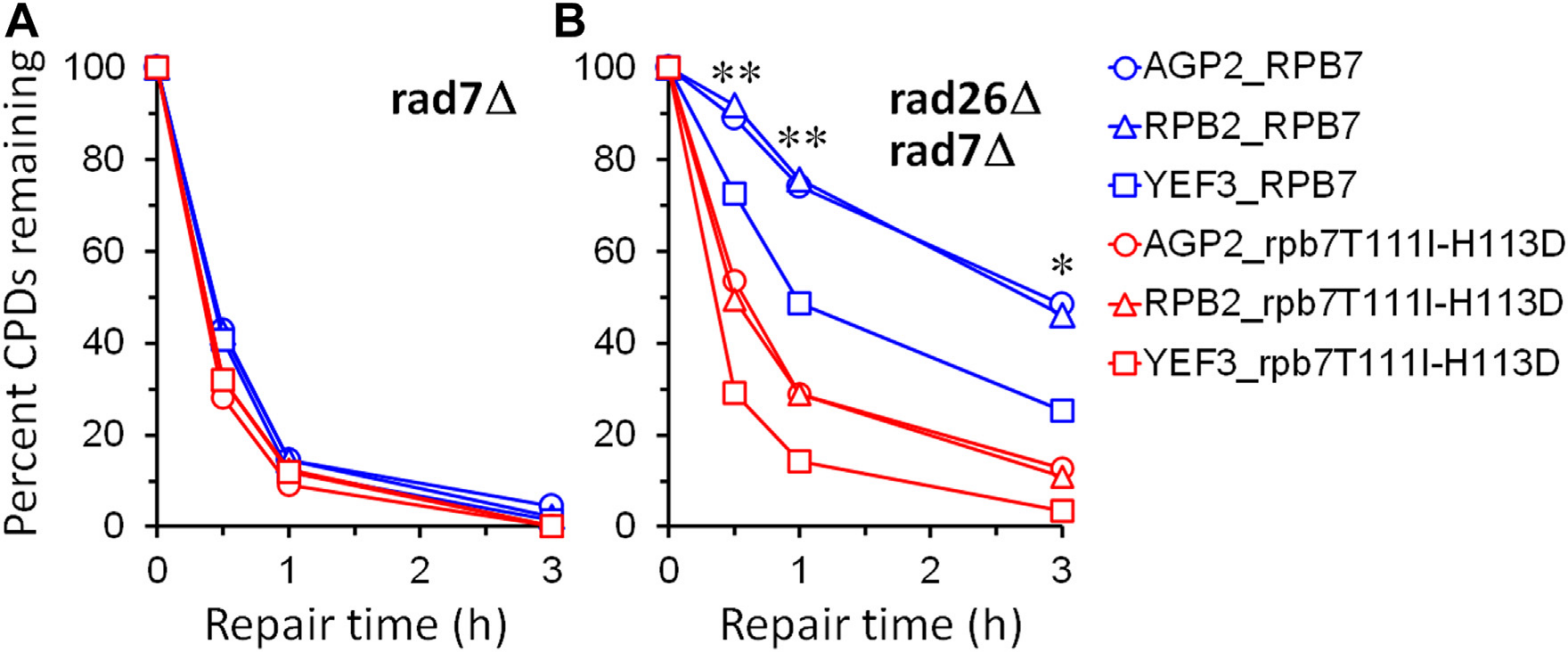
**Plots showing effects of rpb7T111I-H113D on TCR.***A* and *B*, means of percent CPDs remaining at all CPD sites 50 nucleotides downstream of the major TSS in the TS of *AGP2*, *RPB2,* and *YEF3* genes in cells with the wild type and mutant Rpb7. ∗∗ and ∗ indicate *p* values < 0.01 and 0.05, respectively, at the respective repair time points (paired Student’s *t* test). The values for the wild type and mutant Rpb7 were paired with respect to the *AGP2*, *RPB2,* and *YEF3* genes for the Student’s *t* test.

Unlike rpb7G149D, which is viable in all cell types tested, rpb7T111I-H113D caused lethality to *spt4Δ* cells (not shown). However, like rpb7G149D, rpb7T111I-H113D increased the UV resistance of *rad7Δ rad26Δ* cells ∼10-fold but did not significantly affect the UV resistance of the otherwise wild-type, *rad7Δ* or *rad14Δ* cells (Fig. 6). Also, rpb7T111I-H113D restored TCR in *rad7Δ rad26Δ* cells (Fig. 7, compare D with A and C, H with E and G, and, and L with I and K; Fig. 8*B*), but did not substantially affect the repair in *rad7Δ* cells (Fig. 7, compare A and B, E and F, and I and J; Fig. 8*A*). This indicates that, like rpb7G149D, rpb7T111I-H113D derepresses TCR in the absence of Rad26 but does not affect the repair if Rad26 is present.

### Rpb7 regions that interact with the core RNAPII and/or Rpb4 repress TCR synergistically with Spt4

To determine whether Rpb7 regions that interact with the core RNAPII and/or Rpb4 also play roles in regulating TCR, we created and characterized additional Rpb7 mutants (Table 1). As mutation of a single Rpb7 residue, except for G149, may not disrupt the Rpb7 structure extensively enough to dramatically affect UV sensitivity and/or TCR, most of the additional mutants contain changes of multiple residues located on different surface areas of the Rpb7 RNP (rpb7RNP-1 to -12) and/or OB (rpb7OB-1 to -18) domains. Mutations of the N-terminal residues 2 to 5 (rpb7RNP-10) or deletion of 11 residues (161–171) from the C-terminal (rpb7OB-16) caused cell lethality (Table 1). Deletion of residues 65 to 90 (rpb7RNP-7), which span portions of the RNP and OB domains, or mutations in the C3-B4 linker and B4 β-strand in the OB domain (rpb7OB-5) also caused cell lethality (Table 1). Certain mutations caused lethality of *rad14Δ* and/or *spt4Δ* cells but not other cell types tested (Table 1).

**Table 1:** Additional Rpb7 mutants

Mutant	Residue mutated/deleted	Mutant location	Mutant interaction with	Fold UV sensitivity and level of TCR enhancement^a^				
				WT	rad7Δ	rad26Δ rad7Δ	spt4Δ rad26Δ rad7Δ	rad14Δ
RNP-1	15–18Δ	RNP (K1/loop)	core_RNAPII	N	N	3 (2)	5 (2)	N
RNP-2	K5S F18A R21W	RNP (A1, K1-loop)	core_RNAPII, Rpb4	N	10	5 (1)	N (0)	N
RNP-3	G66I	RNP (tip loop)	core_RNAPII	-	-	N	N	-
RNP-4	63–67Δ	RNP (tip loop)	core_RNAPII	N	5	3 (2)	lethal	10
RNP-5	K41D 63–67Δ	RNP (tip loop, A2-A3 linker)	core_RNAPII	5	5	10 (2)	lethal	5
RNP-6	Y44R L62Q N71G	RNP (tip loop, A3)	core_RNAPII	-	N	N	N	-
RNP-7	65–90Δ	RNP (tip loop, A4); OB (B1, B1′)	core_RNAPII, Rpb4, Spt5_KOW3	lethal	lethal	lethal	lethal	lethal
RNP-8	K5S	RNP (A1)	Rpb4	-	N	N	N	-
RNP-9	I4A D6A L7A L9A	RNP (A1)	Rpb4	-	N	N	N	-
RNP-10	F2A F3A I4A K5S	RNP (A1)	Rpb4	lethal	lethal	lethal	lethal	lethal
RNP-11	D6A L26V L30V K41G	RNP (A1, K2, A2-A3 linker)	Rpb4, core_RNAPII	-	N	N	N	-
RNP-12	E33R E35R K41D	RNP (K2, A2-A3 linker)	Rpb4	N	N	N (1)	N (1)	N
OB-1	V86A D88K T90A	OB (B1)	Rpb4	N	N	N (1)	N	N
OB-2	V86A D88K T90A R142A I143D R144A	OB (B1, B4)	Rpb4	-	10	3	lethal	lethal
OB-3	V86A D88K T90A R142A I143D R144A 167–171Δ	OB (B1, B4, B6)	Rpb4	-	20	5 (1)	lethal	lethal
OB-4	140–144Δ	OB (C3-B4 linker, B4)	Rpb4	-	10	10	lethal	lethal
OB-5	K140A R142A I143D R144A K146A	OB (C3-B4 linker, B4)	Rpb4	lethal	lethal	lethal	lethal	lethal
OB-6	R142A I143D R144A	OB (B4)	Rpb4	N	5	5 (0)	20 (2)	lethal
OB-7	142–144Δ	OB (B4)	Rpb4	-	5	3	lethal	lethal
OB-8	R142A R144A K146A E148A	OB (B4)	Rpb4, Spt5_KOW3	N	N	N	N	-
OB-9	I143D 140–141Δ	OB (B4)	Rpb4	N	3	N	N	5
OB-10	I143D Q153Y L168Y A170Y	OB (B4, B4′, B6)	Rpb4, Spt5_KOW3	N	N	5 (3)	30 (2)	N
OB-11	R142A I143D R144A 167–171Δ	OB (B4, B6)	Rpb4	-	10	5 (0)	lethal	lethal
OB-12	151D I160D	OB (B4′, B5)	Spt5_KOW3	-	3	N	lethal	lethal
OB-13	Q153A H158A	OB (B4′, B5)	Spt5_KOW3, KOW4	N	3	N	N	8
OB-14	Q153Y	OB (B4′)	Spt5_KOW3, KOW4	N	N	N	N	8
OB-15	I157A	OB (B5)	?	-	N	N	N	-
OB-16	161–171Δ	OB (B5-B6)	Rpb4	lethal	lethal	lethal	lethal	lethal
OB-17	162–171Δ	OB (B5-B6)	Rpb4	N	N	N (2)	lethal	20
OB-18	167–171Δ	OB (B6)	Rpb4	N	N	N (0)	N	10

a Fold UV sensitivities were at 120, 40, 20, 40 and 6 J/m^2^ for WT, rad7Δ, rad7Δ rad26Δ, rad7Δ rad26Δ spt4Δ, and rad14Δ cells respectively. Numbers in parentheses indicate levels of TCR enhancement. N, UV sensitivity unchanged. -, untested.

**Figure 9:**
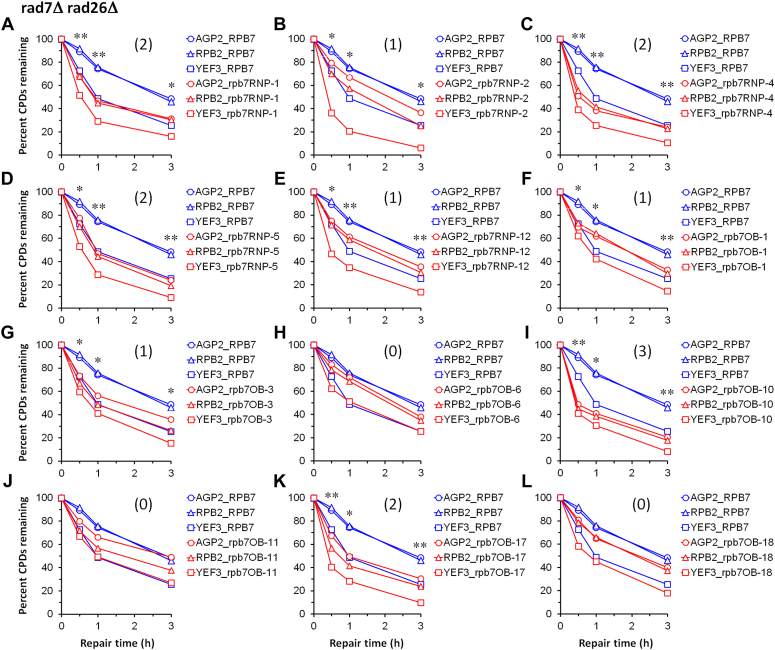
**Plots showing effects of additional Rpb7 mutants on TCR in *rad7Δ rad26Δ* cells.***A*–*L*, means of percent CPDs remaining at all CPD sites 50 nucleotides downstream of the major TSS in the TS of *AGP2*, *RPB2,* and *YEF3* genes in cells with the wild type or indicated mutant Rpb7. ∗∗ and ∗ indicate *p* values < 0.01 and 0.05, respectively, at the respective repair time points (paired Student’s *t* test). The values for the wild type and mutant Rpb7 were paired with respect to the *AGP2*, *RPB2*, and *YEF3* genes for the Student’s *t* test. The number within parenthesis of each panel indicates the level of TCR enhancement by the Rpb7 mutant. Levels 3, 2, 1 and 0, respectively indicate ≥30%, ≥20%, ≥10%, and ≤10% reduction of average percent CPDs remaining for all the genes (*AGP2*, *RPB2*, and *YEF3*) at all repair time points (0.5, 1 and 3 h).

**Figure 10:**
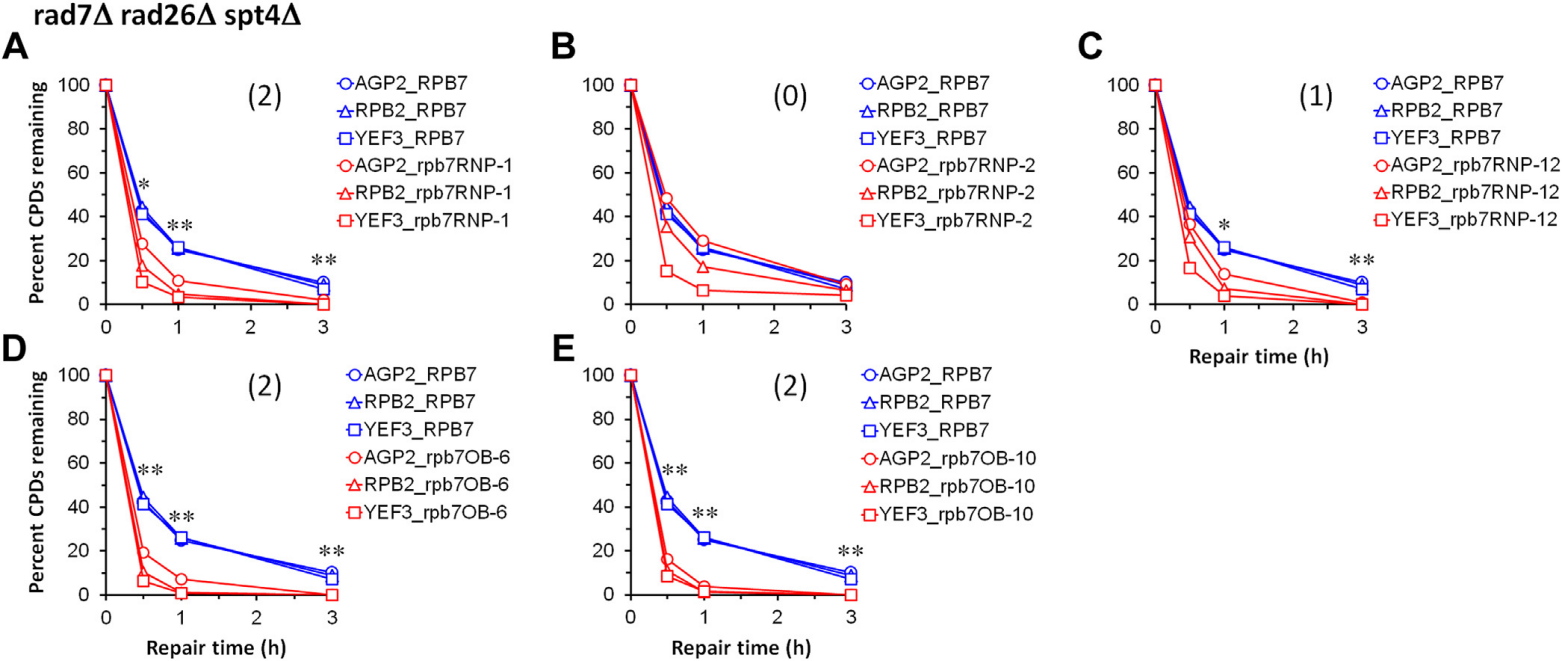
**Plots showing effects of additional Rpb7 mutants on TCR in *rad7Δ rad26Δ spt4Δ* cells.***A*–*E*, means of percent CPDs remaining at all CPD sites 50 nucleotides downstream of the major TSS in the TS of *AGP2*, *RPB2* and *YEF3* genes in cells with the wild type or indicated mutant Rpb7. ∗∗ and ∗ indicate *p* values < 0.01 and 0.05, respectively, at the respective repair time points (paired Student’s *t* test). The values for the wild type and mutant Rpb7 were paired with respect to the *AGP2*, *RPB2*,and *YEF3* genes for the Student’s *t* test. The number within the parenthesis of each panel indicates the level of TCR enhancement by the Rpb7 mutant. Levels 2, 1, and 0, respectively indicate ≥20%, ≥10% and ≤10% reduction of average percent CPDs remaining for all the genes (*AGP2*, *RPB2*, and *YEF3*) at the repair time points of 0.5 and 1 h. The repair timepoint of 3 h was excluded for assessing the level of TCR enhancement because TCR at this time point was close to completion in cells with either the mutant or wild-type Rpb7.

A fraction of the additional Rpb7 mutants caused different levels of UV sensitivity for otherwise wild type, *rad7Δ*, *rad7Δ rad26Δ*, *rad7Δ rad26Δ spt4Δ* and *rad14Δ* cells (Table 1 and Figs. S3–S7). However, none of the additional Rpb7 mutants caused UV resistance of any cell types examined, including *rad7Δ rad26Δ* cells. The UV sensitivity phenotypes of the additional Rpb7 mutants are different from those of rpb7G149D or rpb7T111I-H113D, which conferred *rad7Δ rad26Δ* cells UV resistance (Figs. 2*C* and 6*C*) but did not significantly affect the UV sensitivities of otherwise wild-type, *rad7Δ* or *rad14Δ* cells (Figs. 2 and 6). We, therefore, suspected that some of the additional Rpb7 mutations might attenuate rather than derepress TCR. Surprisingly, regardless of their levels of UV sensitivity, all of the additional Rpb7 mutants that may severely disrupt the interaction of Rpb7 with either the core RNAPII (rpb7RNP-1, -4 and -5, which have K1-loop or tip loop deletion) or Rpb4 (rpb7RNP-12, rpb7OB-1, -3, -10 and -17), or with both the core RNAPII and Rpb4 (rpb7RNP-2) derepressed TCR to certain extents in *rad7Δ rad26Δ* cells (Figs. 9 and S8–S10) but did not affect TCR in *rad7Δ* cells (Figs. S11–S14). The derepression of TCR in *rad7Δ rad26Δ* cells by these additional Rpb7 mutants was generally less extensive than that by rpb7G149D or rpb7T111I-H113D (compare panels in Fig. 9 with Figs. 4*B* and 8*B*). In *rad7Δ rad26Δ spt4Δ* cells, all of the additional Rpb7 mutants analyzed, including rpb7OB-6, which did not significantly affect TCR in *rad7Δ rad26Δ* cells (Fig. 9*H*), significantly enhanced TCR (Figs. 10 and S15–S17). Although the effect of rpb7RNP-2 on TCR in *rad7Δ rad26Δ spt4Δ* cells was statistically insignificant if the *AGP2*, *RPB2* and *YEF3* genes were all considered and analyzed collectively (Fig. 10*B*), the rpb7 mutant substantially enhanced TCR in the rapidly transcribed *YEF3* gene (Fig. 10*B*). These results indicate that Rpb7 regions that interact with the core RNAPII and/or Rpb4 act synergistically with Spt4 in repressing TCR.

None of the additional Rpb7 mutants examined appeared to significantly affect the repair of CPDs in the NTS of the *AGP2*, *RPB2*, and *YEF3* genes (Figs. S18 and S19), indicating that they may not be implicated in GGR. However, in view of the facts that the additional Rpb7 mutants may confer UV sensitivity, rather than UV resistance, to the cells including *rad7Δ rad26Δ* cells, the Rpb7 regions that interact with the core RNAPII and/or Rpb4 may be implicated in other (non-NER) DNA damage repair and/or tolerance mechanism(s) that remain(s) to be elucidated.

## Discussion

Through screening of random mutations and site-directed mutagenesis, we identified multiple Rpb7 mutants that derepress TCR in *rad26Δ* and/or *rad26Δ spt4Δ* cells. Our results indicate that Rpb7 is another TCR repressor, and it may act through the same common mechanism as or synergistically with Spt4/Spt5 in repressing TCR but does not significantly affect GGR.

Spt4 interacts with the Spt5 NGN domain, which binds to the “clamp” of the core RNAPII (Fig. 5*A*). Spt4 indirectly suppresses TCR by protecting Spt5 from degradation and stabilizing the interaction of Spt5 with RNAPII (24). Rad26 (27) and its human homolog CSB (28) contact upstream DNA of the RNAPII elongation complex and clash with Spt4/Spt5 for binding to RNAPII engaged in transcription elongation. Also, the yeast Rad26 (29) and human CSB (28) displace Spt5, which explains how Rad26 antagonizes the repression of TCR by Spt4/Spt5. Our results here indicate that Rpb7 regions that interact with Spt5 KOW3, the core RNAPII, and Rpb4 all contribute to the repression of TCR. The Rpb7 “tip,” consisting of the K1-loop and tip loop (Fig. 5, *A* and *B*), ‘wedges’ the clamp of RNAPII to the closed conformation, resulting in a narrower central cleft of the polymerase (16, 17). Rpb7 mutations located in regions that interact with Spt5 KOW3, the core RNAPII and Rpb4 may all “loosen” the central cleft of RNAPII. Therefore, it appears that the transcription elongation complex is intrinsically repressive for TCR and that certain form of “loosening” of the complex, either by Rad26 displacing Spt4/Spt5 or through disrupting interactions among other components of the RNAPII elongation complex may result in the relaxation of TCR repression.

rpb7G149D or rpb7T111I-H113D, which may closely interact with Spt5 KOW3 (Fig. 5*B*), conferred UV resistance to *rad7Δ rad26Δ* cells but not to *rad7Δ* or *rad14Δ* cells (Figs. 2 and 6). Similarly, *spt4Δ* (24, 25), or truncation of the CTR (24) or the KOW4-KOW5 (45) domains of Spt5, causes UV resistance of *rad7Δ rad26Δ* (*rad16Δ rad26Δ*) cells but does not affect UV sensitivities of other cell types. It is therefore likely that the Spt4/Spt5 complex, and its interaction (through KOW3) with Rpb7 is solely involved in the repression of TCR but plays no role in other DNA damage repair or tolerance mechanisms.

Rpb7 mutants implicated in interactions with the core RNAPII and/or Rpb4 could significantly enhance TCR in *rad7Δ rad26Δ spt4Δ* cells, even if the *AGP2*, *RPB2,* and *YEF3* genes were all considered and analyzed collectively (Fig. 10). The synergism between these Rpb7 mutants and *spt4Δ* is stronger than that between rpb7G149D with *spt4Δ*, as rpb7G149D only mildly enhanced TCR in the *YEF3*, but not *AGP2* or *RPB2* gene in *rad7Δ rad26Δ spt4Δ* cells (Fig. 4*C*). Therefore, while rpb7G149D and possibly rpb7T111I-H113D (which is synthetically lethal with *spt4Δ*) may derepress TCR primarily by disrupting/remodeling interactions with Spt4/Spt5 (*via* Spt5 KOW3), the other Rpb7 mutants implicated in interactions with the core RNAPII and/or Rpb4 may derepress TCR largely independently of Spt4/Spt5.

Despite being able to derepress TCR, none of the Rpb7 mutants implicated in interactions with the core RNAPII and/or Rpb4 caused *rad7Δ rad26Δ* cells UV resistance. Rather, they may or may not make the cells UV sensitive (Table 1 and Figs. S3–S7). This indicates that, in addition to repressing TCR, the Rpb7 regions involved in interactions with the core RNAPII and/or Rpb4 may play positive roles in other (non-NER) DNA damage repair and/or tolerance mechanisms. In these Rpb7 mutant cells, the survival benefit caused by derepression of TCR may be offset or even outweighed by the survival impediment caused by impairment of the other DNA damage repair and/or tolerance mechanisms. We previously found that *rpb4Δ* also derepresses TCR and makes *rad16Δ* cells UV sensitive (20). In contrast to the Rpb7 mutants, *rpb4Δ* does not affect UV sensitivities of other cell types, including the otherwise wild type, *rad16Δ rad26Δ* or *rad1Δ* cells (20). Therefore, the role of Rpb7 in the other DNA damage repair and/or tolerance mechanisms may be more pleiotropic than Rpb4. How Rpb7 functions together with or independently of Rpb4 in regulating the other DNA damage repair and/or tolerance mechanisms remains to be elucidated. One possibility is that Rpb7 itself, or along with Rpb4, may affect transcriptional bypass of DNA lesions, a DNA damage tolerance mechanism that may enhance cell survival (29, 47, 48, 49). It is also possible that Rpb7 itself, or along with Rpb4, may be involved in resolving the collision between the DNA transcription and replication machineries, thereby regulating translesion DNA synthesis, another DNA damage tolerance mechanism that enhances cell survival (50, 51, 52). Furthermore, in view of the fact that Rpb7 is an essential RNAPII subunit, it is also possible that Rpb7 may be required for transcription of certain essential genes especially after UV irradiation. Despite being able to derepress TCR, certain Rpb7 mutations may severely affect transcription of the essential genes, leading to UV sensitivity.

Why *rad26Δ* slows down TCR less severely in the rapidly transcribed *YEF3* gene than in the slowly/moderately transcribed *AGP2* or *RPB2* gene (Fig. 4, *A* and *B*, compare symbols in blue) remains to be elucidated. Transcription elongation is finely tuned by dozens of regulatory factors, including transcription elongation factors, chromatin modifiers, and remodelers (53). The stoichiometric composition of the RNAPII complex engaged in rapid transcription may exert less repression of TCR than that engaged in slow transcription. If that is the case, the rapidly transcribed genes should be less dependent on Rad26 for antagonizing TCR repressors. The less repression of TCR may also explain why certain Rpb7 mutants, such as rpb7RNP-2 and rpb7G149D, significantly enhanced TCR only in the *YEF3* gene but not in the *AGP2* or *RPB2* gene in *rad7Δ rad26Δ spt4Δ* cells (Figs. 4*C* and 10*B*). Future quantitative analyses of the stoichiometric composition of RNAPII complexes engaged in slowly and rapidly transcribed genes will be needed to elucidate if/how TCR is differently repressed in these genes.

## Experimental procedures

### Yeast strains and plasmids

All yeast strains used in this study were derivatives of BJ5465 (MATa *ura3-52 trp1 leu2Δ1 his3Δ200 pep4::HIS3 prb1Δ1.6R can1*) (54). Plasmid pRS416-RPB7 was created by inserting the whole *RPB7* gene including the promoter, coding sequence, and 3′ terminator sequences between the EagI and BamHI sites of pRS416, which bears *URA3* as its selection marker (55). pRS415-RPB7PX was created by inserting the *RPB7* gene fragment, including the native promoter and coding regions of the gene, between the EagI and XhoI sites of pRS415, which has *LEU2* as its selection marker (55). Plasmid pLH157, which contains the genetically engineered *E. coli* tRNA_CUA_ and tyrosyl-tRNA synthetase genes, was obtained from Dr Steven Hahn. pRPB7Bpa plasmids encoding Rpb7 with its E100, E148, I151, H158, or I160 replaced with Bpa were created as described previously (45). Plasmids pRPB7Bpa-G149D and pRPB7Bpa-T111I-H113D were generated by introducing G149D and T111I-H113D mutations, respectively, into the pRPB7Bpa plasmids.

### Screening of Rpb7 random mutants

The screening strategy is outlined in Figure 1. A pool of *RPB7* gene molecules randomly mutagenized in the region of codons 2 to 171 was prepared by error-prone PCR using a GeneMorph II Random Mutagenesis Kit (Agilent). A plasmid library of Rpb7 mutants was created by replacing the *RPB7* gene region of codons 2 to 171 on plasmid pRS415-RPB7PX with the randomly mutated *RPB7* molecules. To maximize the possibility that all 20 possible amino acids can be encoded at each of the 170 (2–171) *RPB7* codons, over 2 million independent *E. coli* transformants were obtained. The library was transformed into *rad7Δ rad26Δ* cells whose genomic *RPB7* gene had been deleted and complemented with pRS416-RPB7 to generate 2 to 3 million independent yeast transformants. The pRS416-RPB7 plasmid was removed from the cells by selection with 5-fluorootic acid (56). Over 1 billion of the 5-FOA selected yeast cells were irradiated with 10 J/m^2^ of UV (254 nm, from a 15 W UV germicidal bulb, General Electric), which kills ∼90% of the *rad7Δ rad26Δ* yeast cells. The UV irradiated and unirradiated cells were allowed to grow for 10 cell divisions to allow the UV sensitive Rpb7 mutants to deplete and the UV resistant ones to enrich. The pRS415 plasmids bearing the randomly mutagenized *RPB7* gene molecules were isolated from the UV-irradiated and unirradiated yeast cells. Three independent repeats, from the transformation of the yeast cells with the plasmid library, UV irradiation, and post-irradiation cell culture to plasmid isolation from the yeast cells, were performed.

The region of the mutagenized *RPB7* gene molecules was sequenced using an Illumina platform (2 × 300 bp). The paired-end sequences for each of the *RPB7* molecules were joined using PANDAseq (57). The counts of *RPB7* codons presented in the UV-irradiated and unirradiated pools were calculated using R packages Biostrings, rtracklayer, GenomicRanges and data.table. The enrichment or depletion of *RPB7* codons was analyzed by using the R package NOISeq, which was designed for the analysis of differential gene expression for RNA-seq data (58). The batch variations of different screening repeats were corrected by utilizing the ARSyNseq function of NOISeq.

To confirm the UV-sensitive or resistant Rpb7 mutants, *LEU2* plasmids bearing the candidate mutants were created and transformed into yeast cells whose genomic *RPB7* gene had been deleted and complemented with pRS416-RPB7. Following the removal of pRS416-RPB7, the UV sensitivity of the cell was analyzed.

### Bpa crosslinking assay

Yeast cells containing pLH157 and pRPB7Bpa, pRPB7Bpa-G149D, or pRPB7Bpa-T111I-H113D were grown in SD medium containing 0.5 mM Bpa (Bachem) to late log phase (OD_600_ ≈ 1.0) and harvested. After harvesting, cells from 15 ml of culture were washed twice with ice-cold H2O, resuspended in 20 ml of ice-cold 2% glucose, and divided into two aliquots. One aliquot was kept on ice, while the other was transferred into a 10-cm diameter glass Petri dish and irradiated with 365 nm UVA for 20 min (total dose of 72,000 J/m^2^) on ice. Following irradiation, cells were pelleted, resuspended in 250 μl of buffer-equilibrated phenol (pH 8.0), and broken by vortexing with 300 μl of glass beads for 15 min. The phenol-cell lysate mixture was transferred to a fresh tube and added with 1.25 ml of methanol containing 0.1 M ammonium acetate. Proteins from the cell lysate were pelleted by centrifugation at 16,000*g* for 15 min at 4 °C, washed with ice-cold 80% acetone, and resuspended in 200 μl of SDS-PAGE gel loading buffer. Rpb7 and Spt5 proteins were probed on Western blots, as previously described (45).

### Mapping repair of UV-induced CPDs

Yeast cells were grown in SD medium at 30 °C to late log phase (OD_600_ ≈ 1.0), irradiated with 120 J/m^2^ of ∼254 nm UV, and incubated in a complete medium in the dark at 30 °C. At different time points of the repair incubation, aliquots were removed, and the genomic DNA was isolated. To analyze CPDs remaining in the *AGP2*, *RPB2* and *YEF3* genes, we adapted the Lesion-Adjoining Fragment Sequencing (LAF-Seq) method, which was originally developed for mapping *N*-methylpurines (59). The genomic DNA was digested with HincII, NruI, NsiI, and HhaI to release the *AGP2*, *RPB2,* and *YEF3* gene fragments, incised at the CPD sites with T4 endonuclease V, and the 3′ phosphate group resulting from T4 endonuclease V incision was removed by treatment with *E. coli* endonuclease IV. The *AGP2*, *RPB2,* and *YEF3* gene fragments were strand-specifically fished out using biotinylated oligonucleotides and streptavidin beads. The 3′ ends of the fished-out fragments were ligated with a common adapter sequence using Circligase (59). The fragments were added with barcoded Illumina sequencing adapters by eight cycles of PCR and sequenced on an Illumina HiSeq platform.

The sequencing reads were aligned to the TS and/or NTS of the *AGP2*, *RPB2,* and *YEF3* genes using Bowtie 2 (60). The numbers of reads from the UV-irradiated samples were normalized to those from the control (unirradiated with UV) samples. Reads corresponding to CPDs at individual sites along the gene fragments were counted after subtraction of the background counts (in the unirradiated samples) by using codes in R. To quantify the extent of CPD induction and repair at individual sites along the TS and/or NTS of the genes, we calculated the percentage of CPDs remaining based on the read counts. To better illustrate the CPD induction and repair profiles, we generated pseudo images using R codes that assigned band intensities corresponding to the counts of aligned sequencing reads. These images allowed us to directly visualize the distribution and magnitude of CPD damage and repair along the gene regions of interest.

### UV sensitivity assay

Yeast cells were cultured to saturation at 30 °C. Sequential 10-fold dilutions of the cultures were made. For spotting assay, 5 μl of the diluted cell suspension was spotted onto plates and irradiated with varying doses of ∼254 nm UV. The plates were incubated in the dark at 30 °C for 3 to 5 days before being photographed. For the colony formation assay, 50 μl of the diluted cell suspension was spread onto plates and irradiated with varying doses of ∼254 nm UV. The plates were incubated in the dark at 30 °C for 3 to 5 days, and the colonies were counted. The survival values given are the means of four independent experiments.

## Data availability

The data underlying this article will be shared on reasonable request to the corresponding author.

## Supporting information

This article contains supporting information.

## Conflict of interest

The authors declare that they have no conflicts of interest with the contents of this article.
