# Supplementary material for: Rpb7 represses transcription-coupled nucleotide excision repair
Source: J Biol Chem. 2023 Jun 27;299(8):104969. doi: 10.1016/j.jbc.2023.104969 (PMC10382679; doi:10.1016/j.jbc.2023.104969)
Supplement: Supporting Figures S1–S19 [file mmc3.pdf]

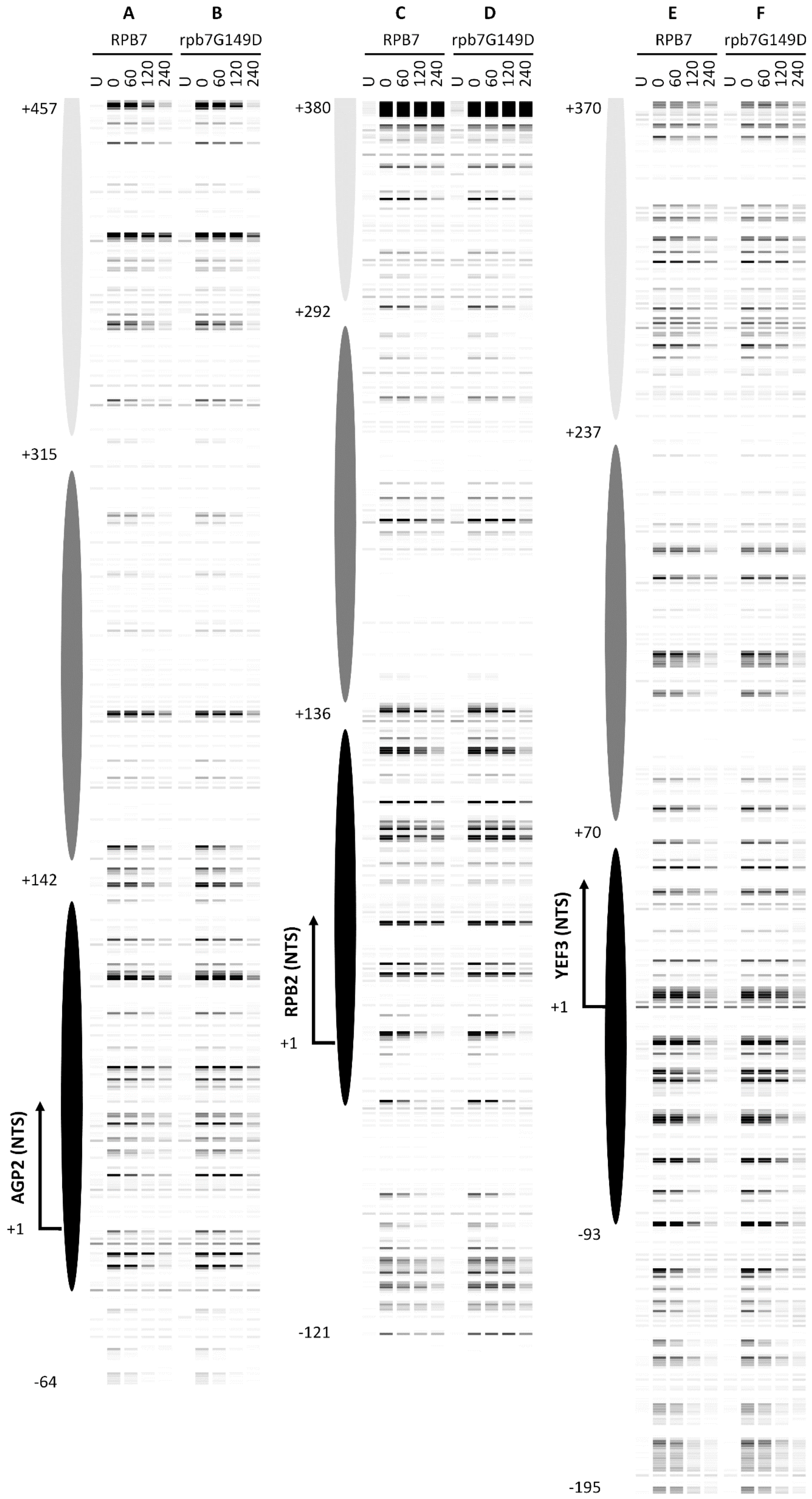


**Supplementary Figure S1.** Pseudo images showing effects of rpb7G149D on GGR. (A-F) Bands reflecting CPD levels along the NTS of *AGP2*, *RPB2* and *YEF3* genes in cells of indicated genotypes at the indicated repair times (min). '*U*', sample unirradiated with UV. Numbers indicating the nucleotide positions of the genes are relative to the major TSS (+1). Ovals indicate positions of nucleosomes, with darkness reflecting adjacency to the TSS.

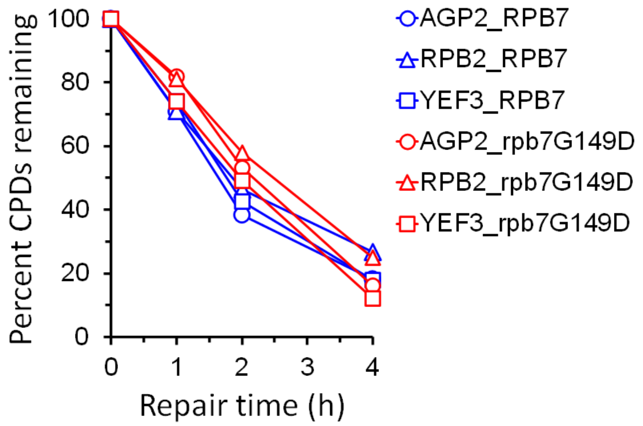


**Supplementary Figure S2.** Plots showing effects of rpb7G149D on GGR. Values are means of percent CPDs remaining at all CPD sites on the NTS of AGP2, RPB2 and YEF3 genes.

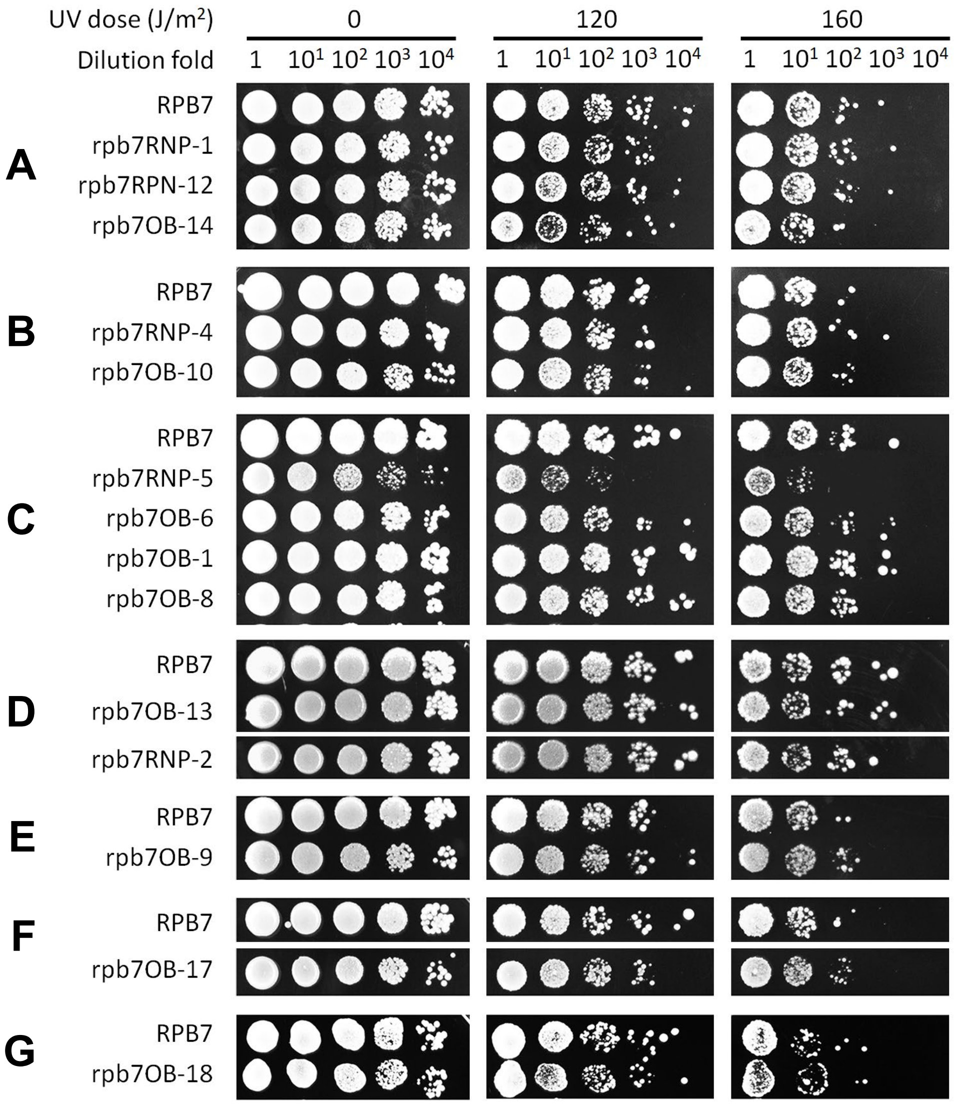


**Supplementary Figure S3.** Effects of additional Rpb7 mutants on UV sensitivity of otherwise wild type cells. Images show spotted yeast cells of indicated genotypes following irradiation with the indicated UV doses. In each panel, RPB7 (wild type) and Rpb7 mutant cells were on the same plate of each of the indicated UV doses. Note that RPB7 cells in panel D were the same as the RPB7 cells in Figure 2A.

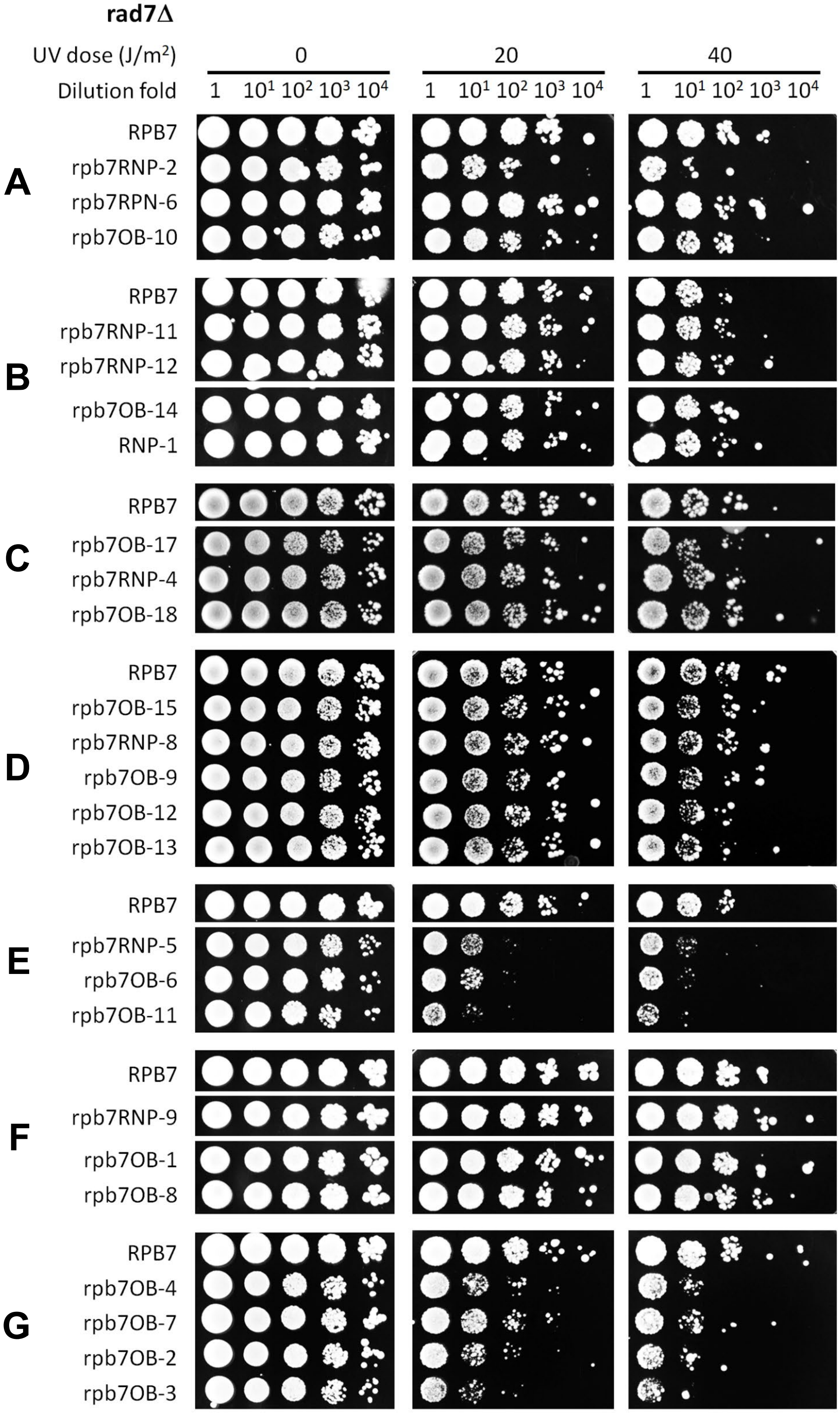


**Supplementary Figure S4.** Effects of additional Rpb7 mutants on UV sensitivity of *rad7Δ* cells. Images show spotted yeast cells of indicated genotypes following irradiation with the indicated UV doses. In each panel, RPB7 (wild type) and Rpb7 mutant cells were on the same plate of each of the indicated UV doses. Note that RPB7 cells in panel E were the same as the RPB7 cells in Figure 2B.

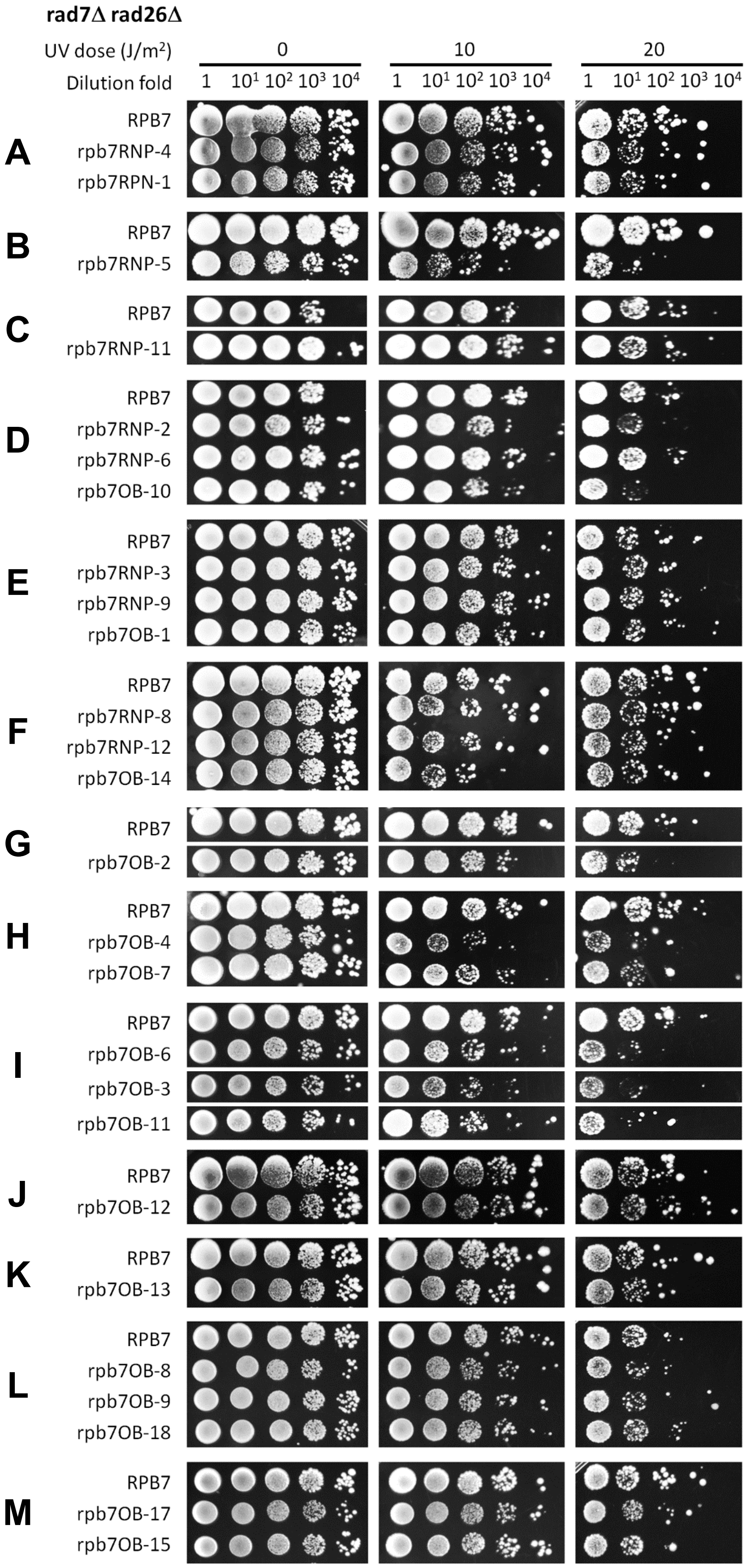


**Supplementary Figure S5.** Effects of additional Rpb7 mutants on UV sensitivity of *rad7Δ rad26Δ* cells. Images show spotted yeast cells of indicated genotypes following irradiation with the indicated UV doses. In each panel, RPB7 (wild type) and Rpb7 mutant cells were on the same plate of each of the indicated UV doses.

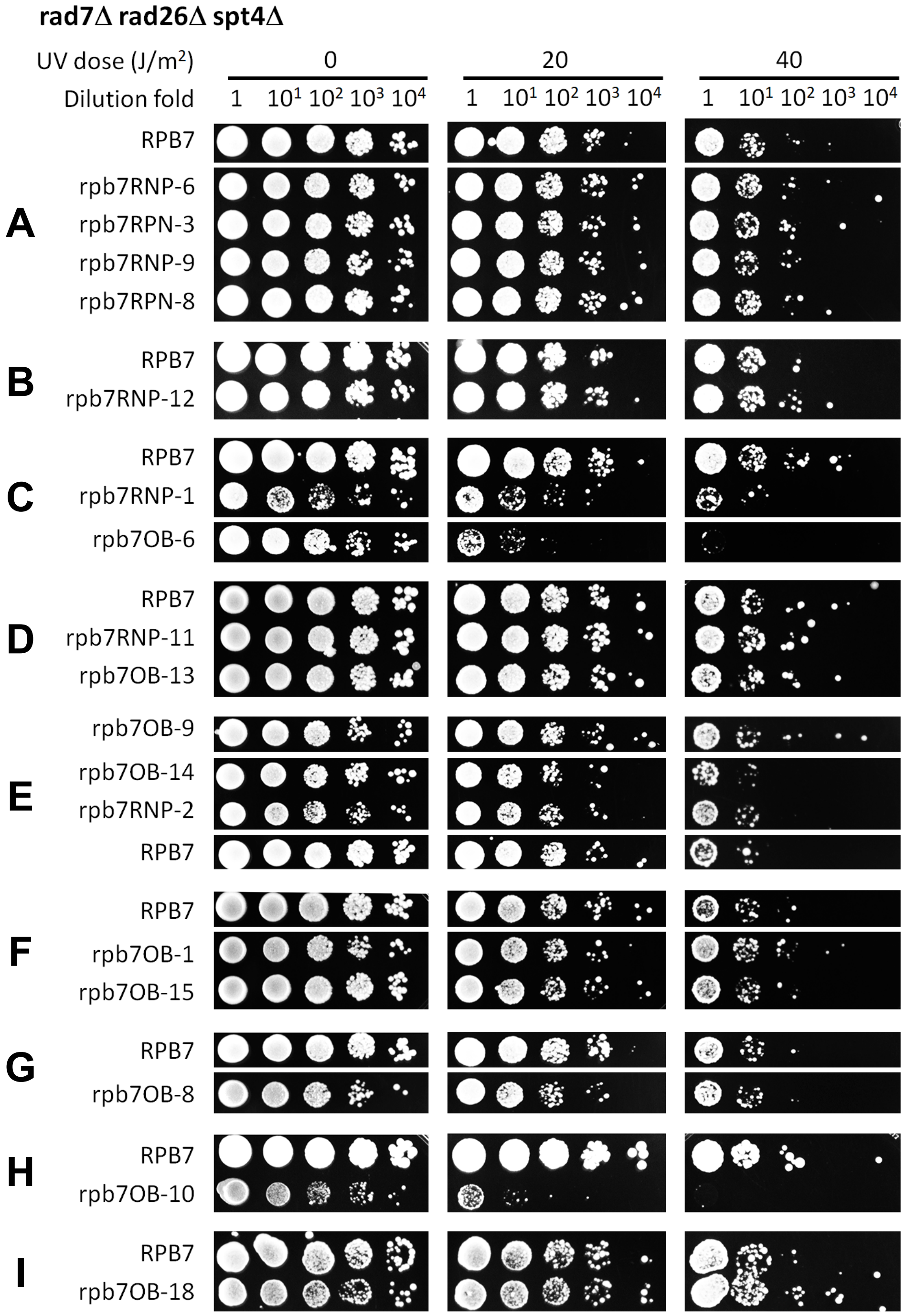


**Supplementary Figure S6.** Effects of additional Rpb7 mutants on UV sensitivity of *rad7Δ rad26Δ spt4Δ* cells. Images show spotted yeast cells of indicated genotypes following irradiation with the indicated UV doses. In each panel, RPB7 (wild type) and Rpb7 mutant cells were on the same plate of each of the indicated UV doses. Note that RPB7 cells in panel D were the same as the RPB7 cells in Figure 2D.

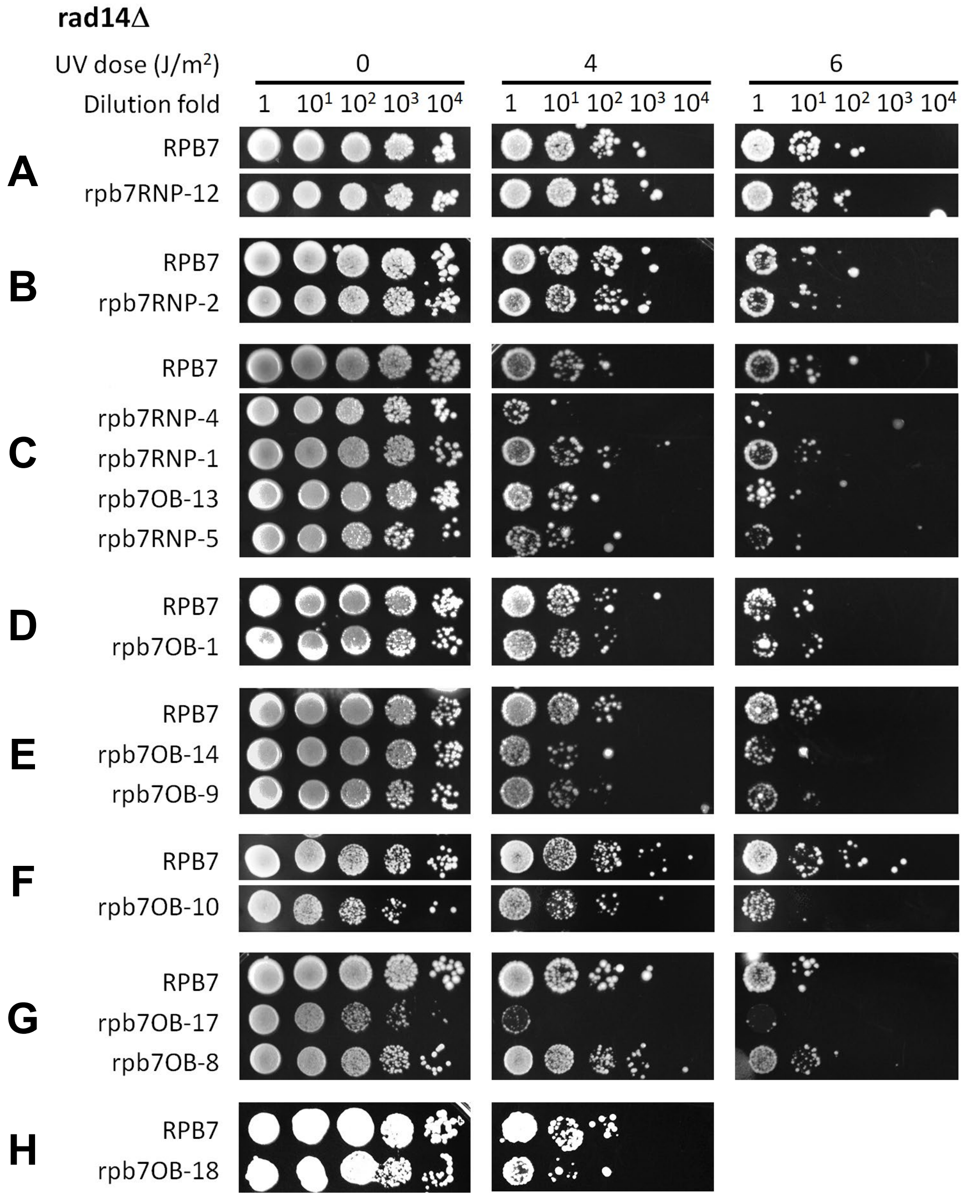


**Supplementary Figure S7.** Effects of additional Rpb7 mutants on UV sensitivity of *rad14Δ* cells. Images show spotted yeast cells of indicated genotypes following irradiation with the indicated UV doses. In each panel, RPB7 (wild type) and Rpb7 mutant cells were on the same plate of each of the indicated UV doses.

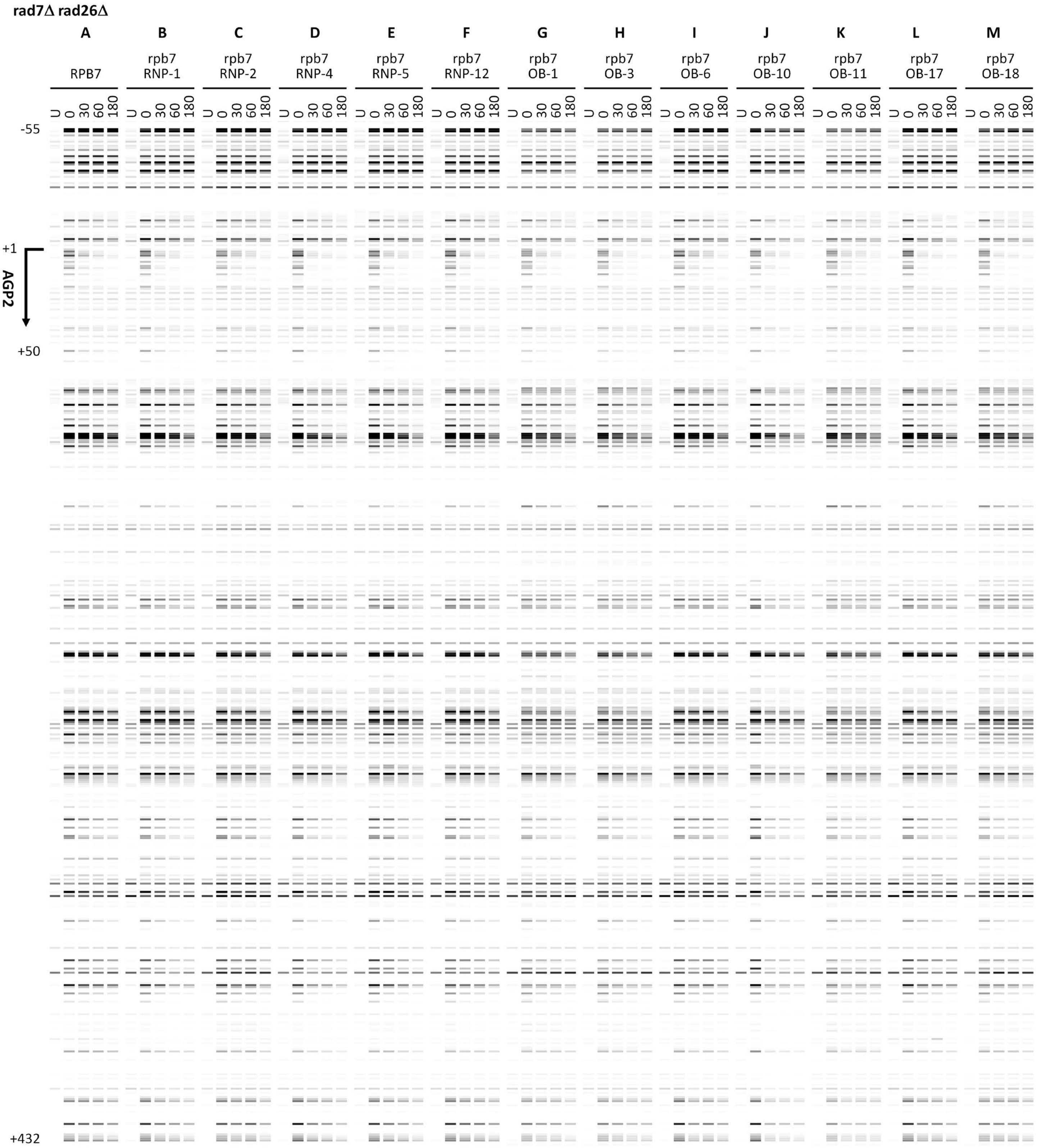


**Supplementary Figure S8.** Pseudo images showing effects of additional Rpb7 mutants on TCR in the *AGP2* gene in *rad7Δ rad26Δ* cells. (A-M) Bands reflecting CPD levels along the TS of *AGP2* genes in *rad7Δ rad26Δ* cells with wild type and mutant Rpb7 at the indicated repair times (min). '*U*', sample unirradiated with UV. Numbers indicating the nucleotide positions of the genes are relative to the major TSS (+1). Note that panel A, which serves as wild type Rpb7 control for comparison with Rpb7 mutants, is the same as Figure 3C.

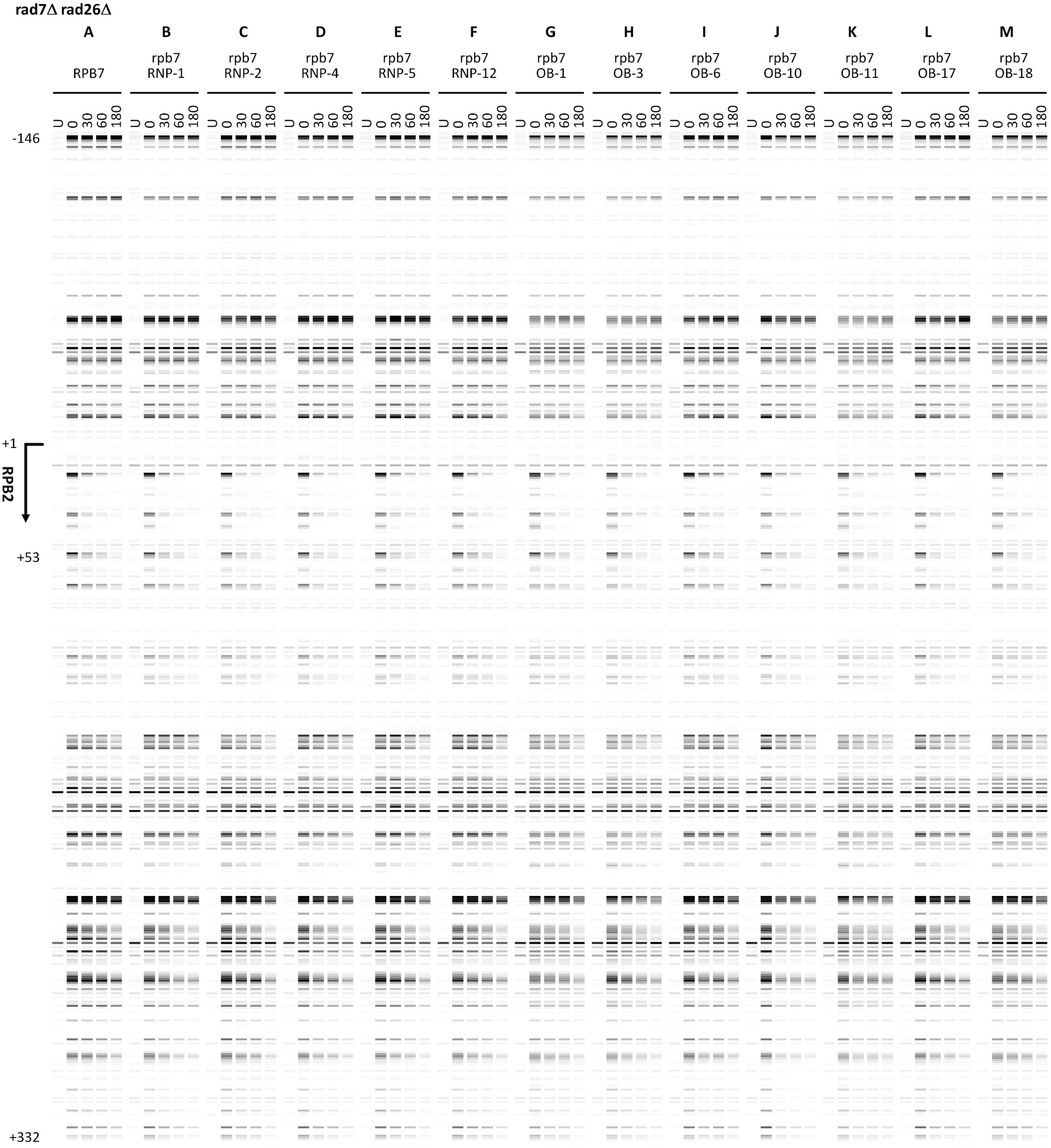


**Supplementary Figure S9.** Pseudo images showing effects of additional Rpb7 mutants on TCR in the *RPB2* gene in *rad7Δ rad26Δ* cells. (A-M) Bands reflecting CPD levels along the TS of *RPB2* genes in *rad7Δ rad26Δ* cells with wild type and mutant Rpb7 at the indicated repair times (min). '*U*', sample unirradiated with UV. Numbers indicating the nucleotide positions of the genes are relative to the major TSS (+1). Note that panel A, which serves as wild type Rpb7 control for comparison with Rpb7 mutants, is the same as Figure 3I.

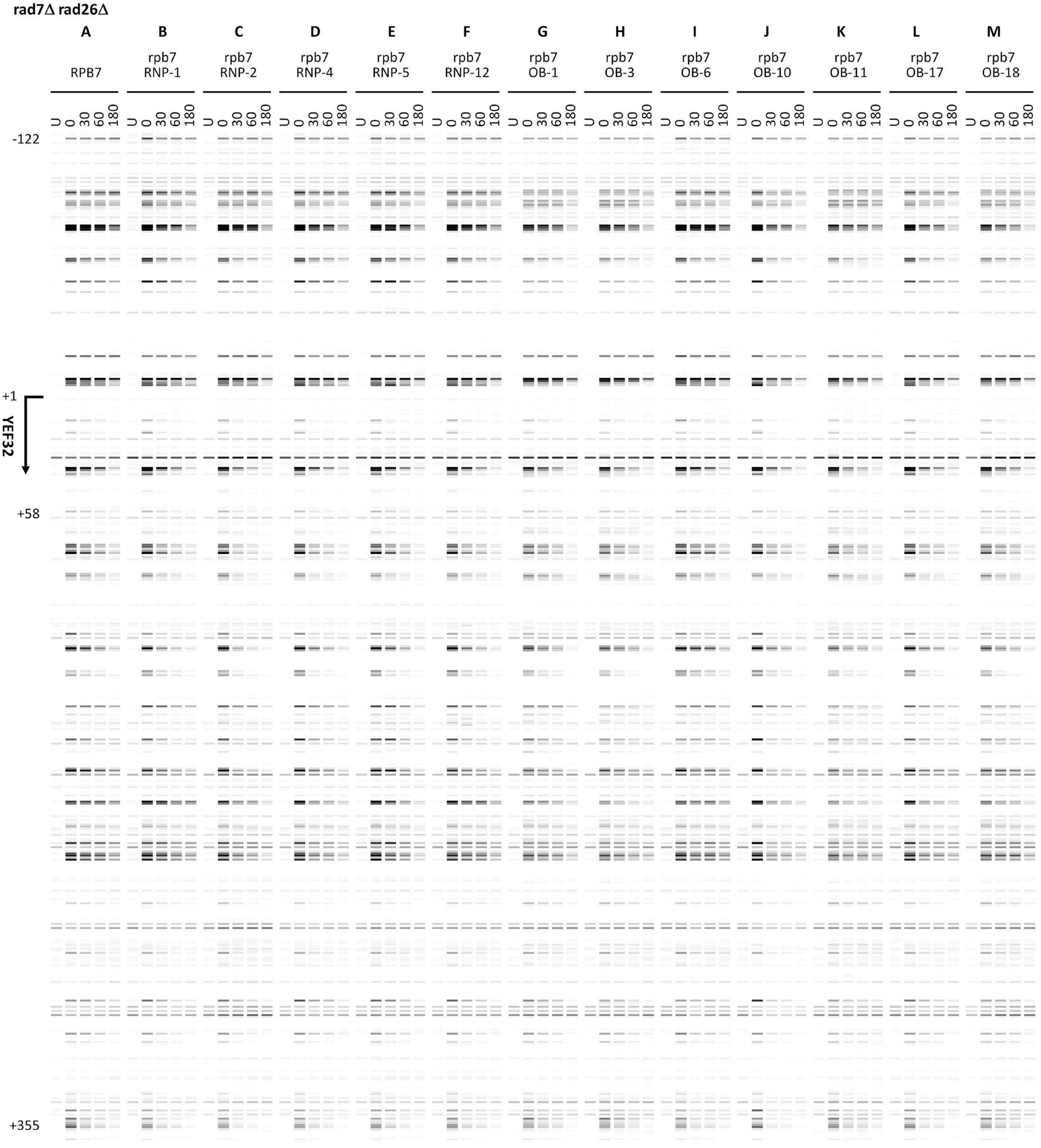


**Supplementary Figure S10.** Pseudo images showing effects of additional Rpb7 mutants on TCR in the *YEF3* gene in *rad7Δ rad26Δ* cells. (A-M) Bands reflecting CPD levels along the TS of *YEF3* genes in *rad7Δ rad26Δ* cells with wild type and mutant Rpb7 at the indicated repair times (min). '*U*', sample unirradiated with UV. Numbers indicating the nucleotide positions of the genes are relative to the major TSS (+1). Note that panel A, which serves as wild type Rpb7 control for comparison with Rpb7 mutants, is the same as Figure 3O.

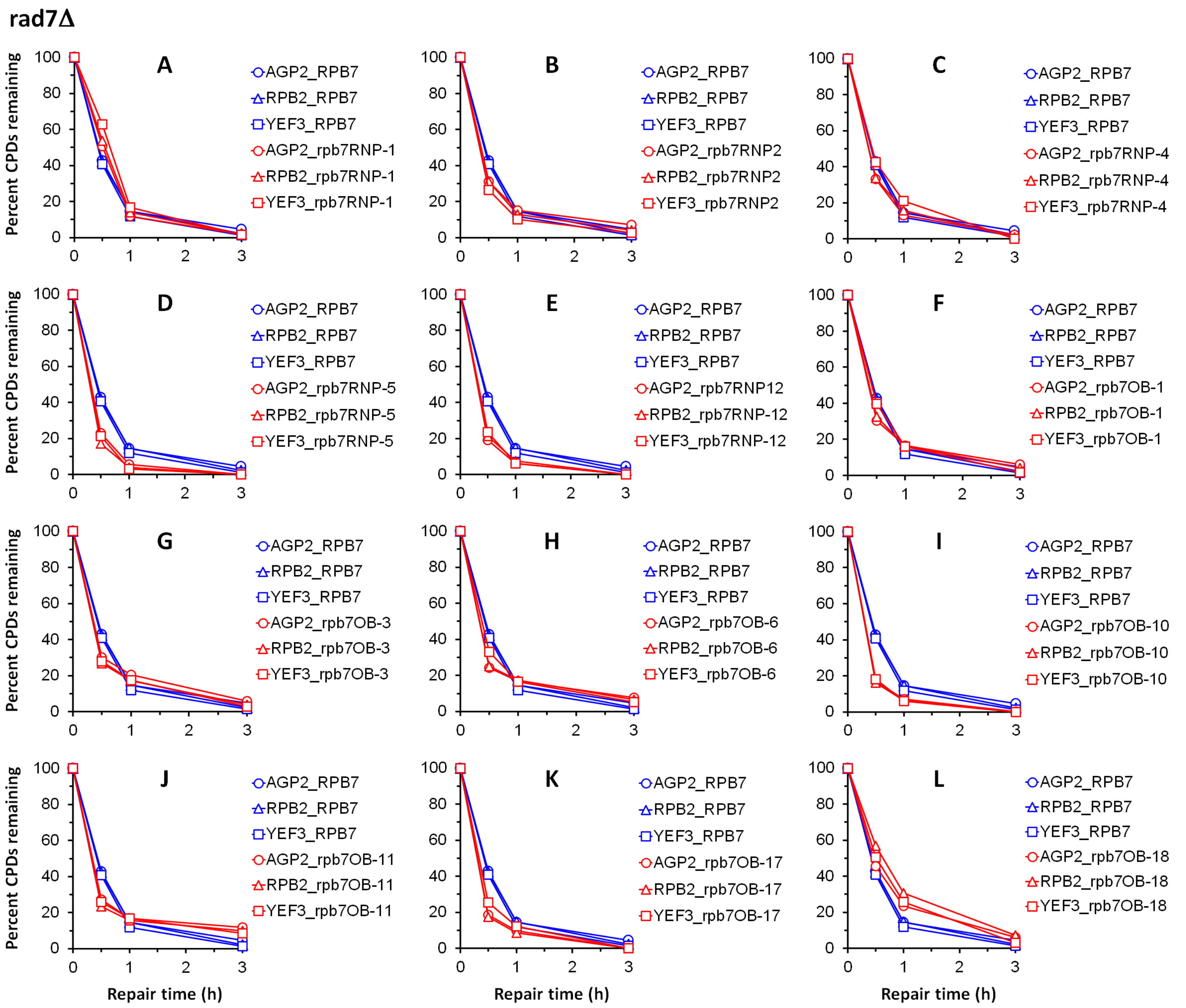


**Supplementary Figure S11.** Plots showing effects of additional Rpb7 mutants on TCR in *rad7Δ* cells. (A-L) Means of percent CPDs remaining at all CPD sites 50 nucleotides downstream of the MAJOR TSS in the TS of *AGP2*, *RPB2* and *YEF3* genes in cells with the wild type or indicated mutant Rpb7.

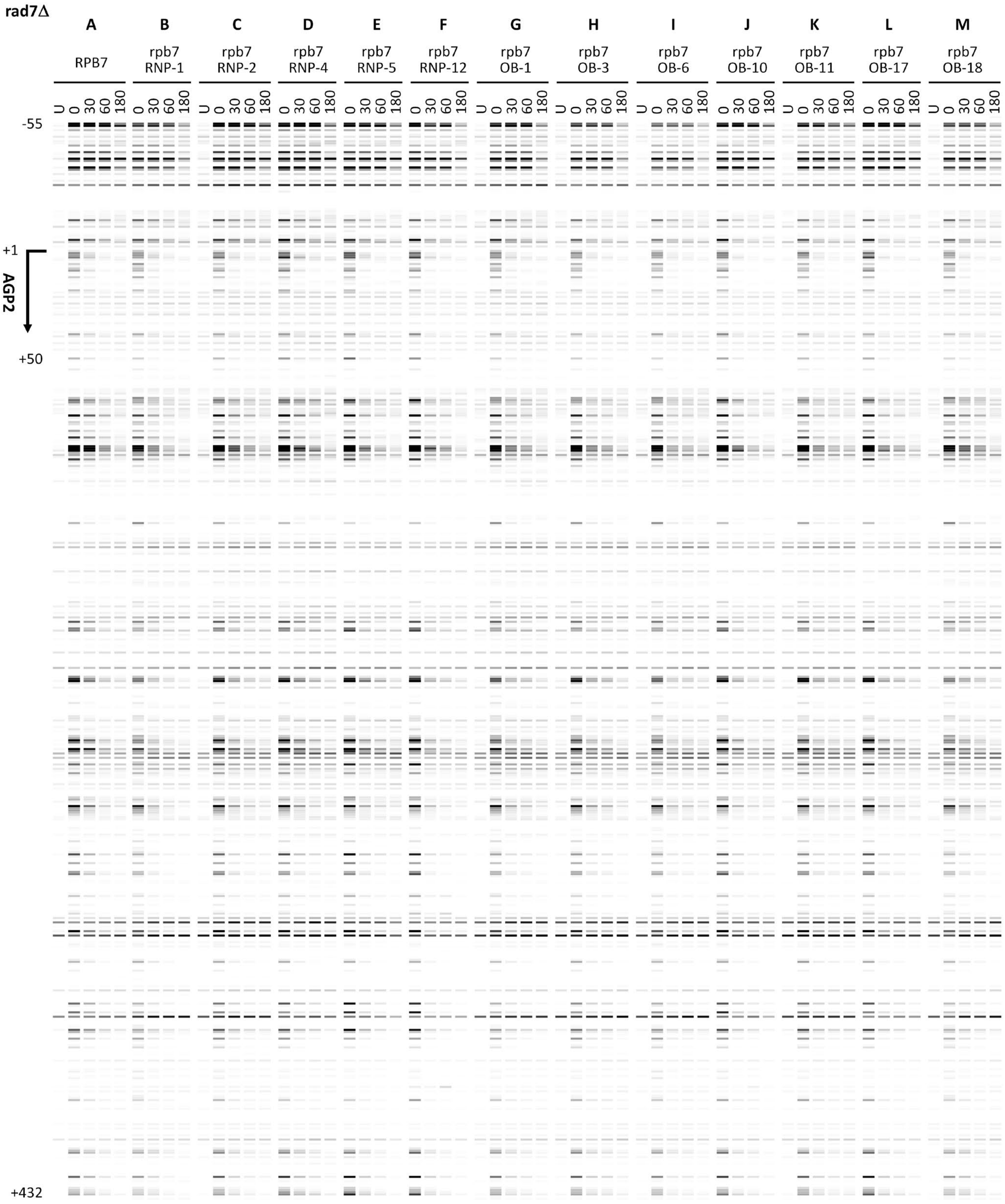


**Supplementary Figure S12.** Pseudo images showing effects of additional Rpb7 mutants on TCR in the *AGP2* gene in *rad7Δ* cells. (A-M) Bands reflecting CPD levels along the TS of *AGP2* genes in *rad7Δ* cells with wild type and mutant Rpb7 at the indicated repair times (min). '*U*', sample unirradiated with UV. Numbers indicating the nucleotide positions of the genes are relative to the major TSS (+1). Note that panel A, which serves as wild type Rpb7 control for comparison with Rpb7 mutants, is the same as Figure 3A.

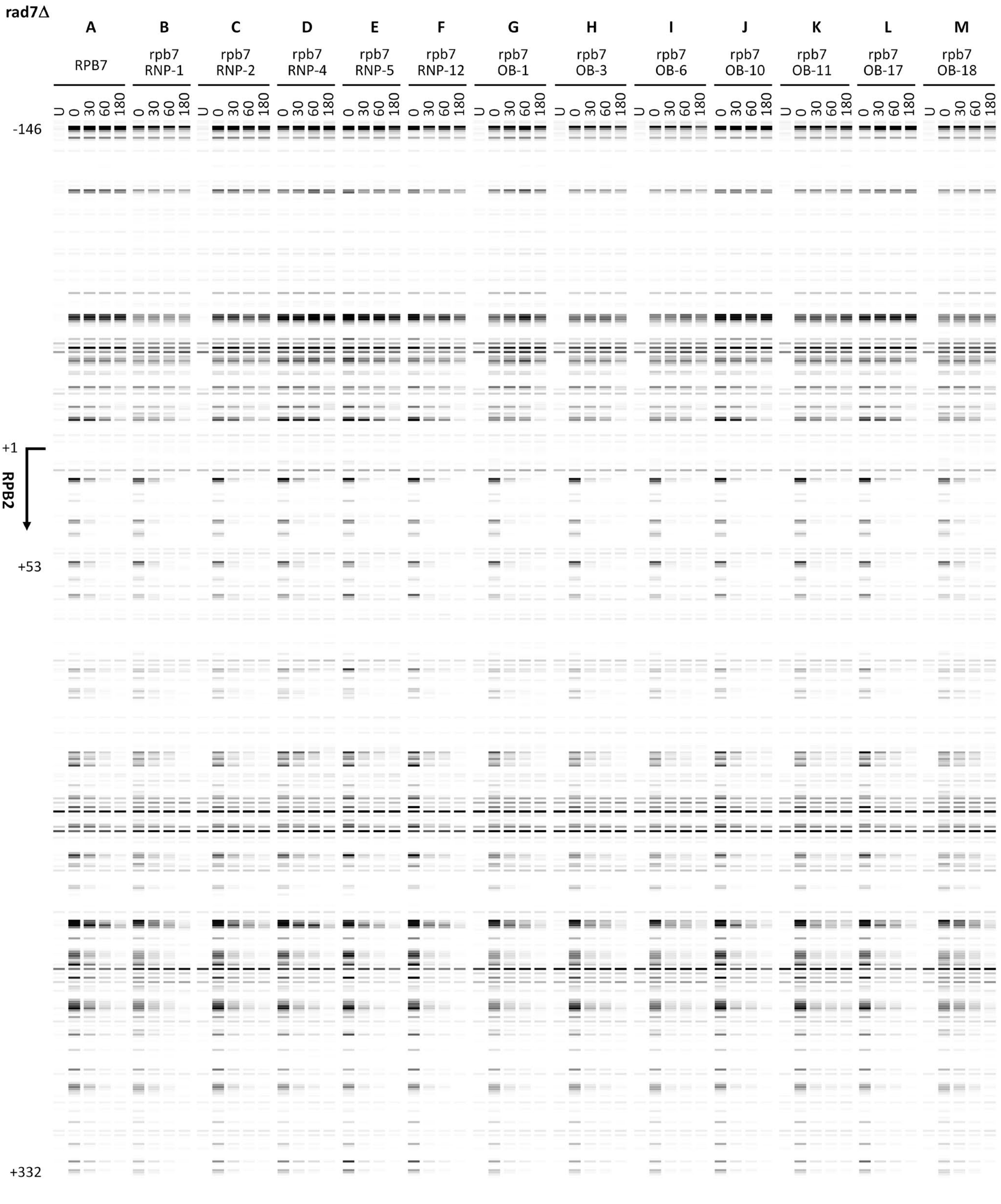


**Supplementary Figure S13.** Pseudo images showing effects of additional Rpb7 mutants on TCR in the *RPB2* gene in *rad7Δ* cells. (A-M) Bands reflecting CPD levels along the TS of *RPB2* genes in *rad7Δ* cells with wild type and mutant Rpb7 at the indicated repair times (min). '*U*', sample unirradiated with UV. Numbers indicating the nucleotide positions of the genes are relative to the major TSS (+1). Note that panel A, which serves as wild type Rpb7 control for comparison with Rpb7 mutants, is the same as Figure 3G.

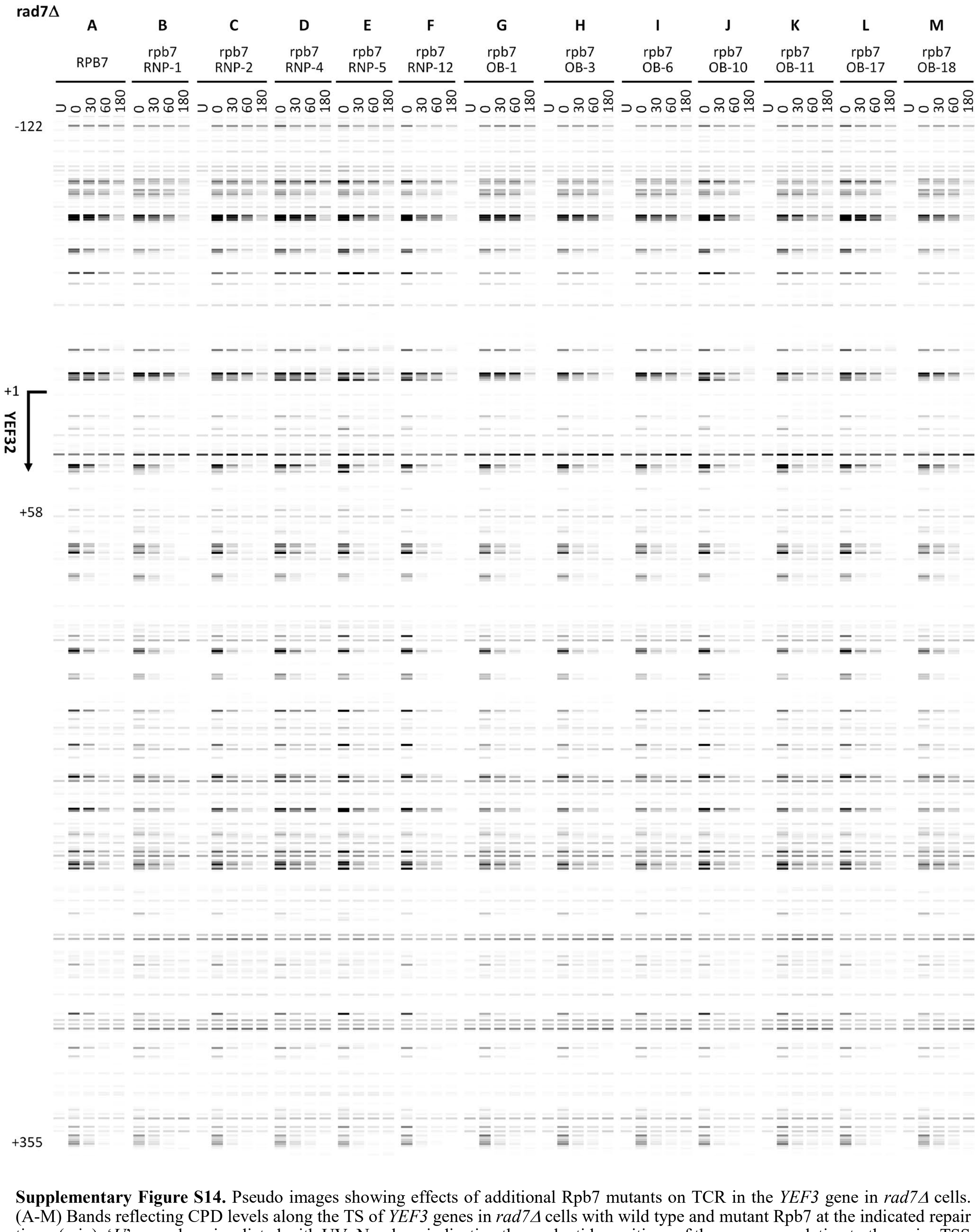


**Supplementary Figure S14.** Pseudo images showing effects of additional Rpb7 mutants on TCR in the *YEF3* gene in *rad7Δ* cells. (A-M) Bands reflecting CPD levels along the TS of *YEF3* genes in *rad7Δ* cells with wild type and mutant Rpb7 at the indicated repair times (min). '*U*', sample unirradiated with UV. Numbers indicating the nucleotide positions of the genes are relative to the major TSS (+1). Note that panel A, which serves as wild type Rpb7 control for comparison with Rpb7 mutants, is the same as Figure 3M.

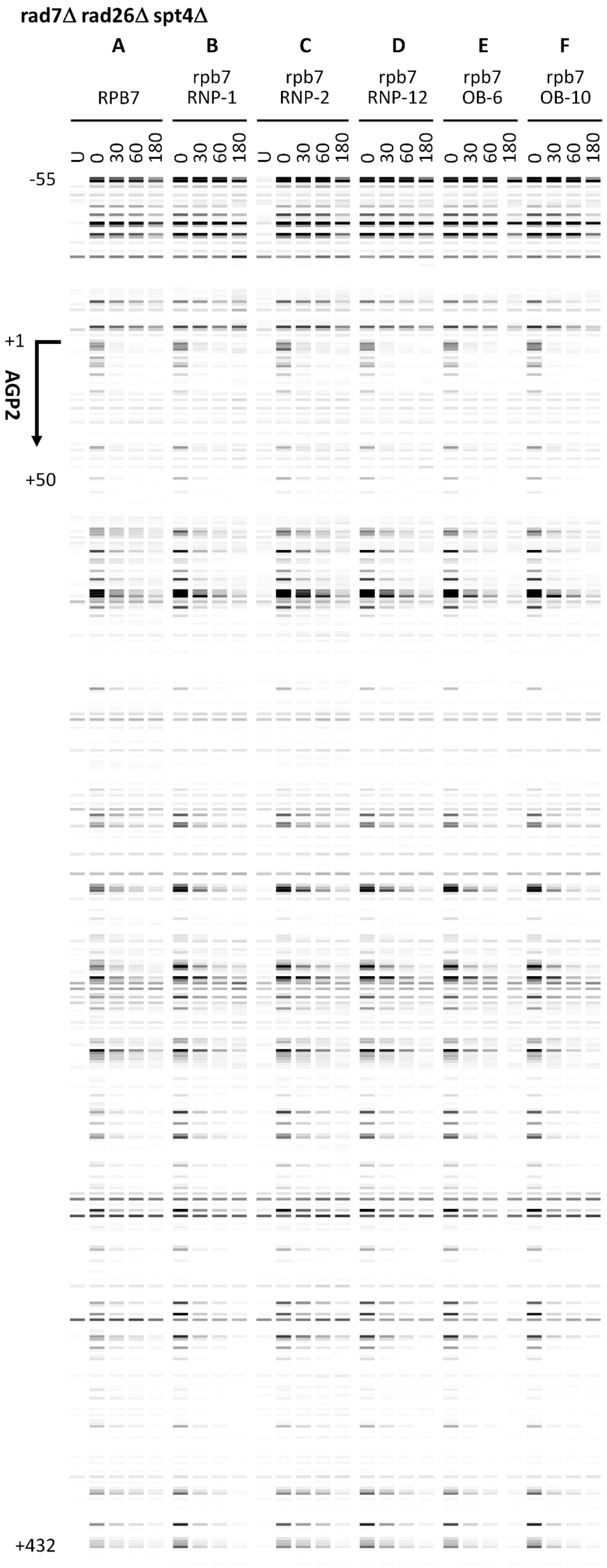


**Supplementary Figure S15.** Pseudo images showing effects of additional Rpb7 mutants on TCR in the *AGP2* gene in *rad7Δ rad26Δ spt4Δ* cells. (A-F) Bands reflecting CPD levels along the TS of *AGP2* genes in *rad7Δ rad26Δ spt4Δ* cells with wild type and mutant Rpb7 at the indicated repair times (min). '*U*', sample unirradiated with UV. Numbers indicating the nucleotide positions of the genes are relative to the major TSS (+1). Note that panel A, which serves as wild type Rpb7 control for comparison with Rpb7 mutants, is the same as Figure 3E.

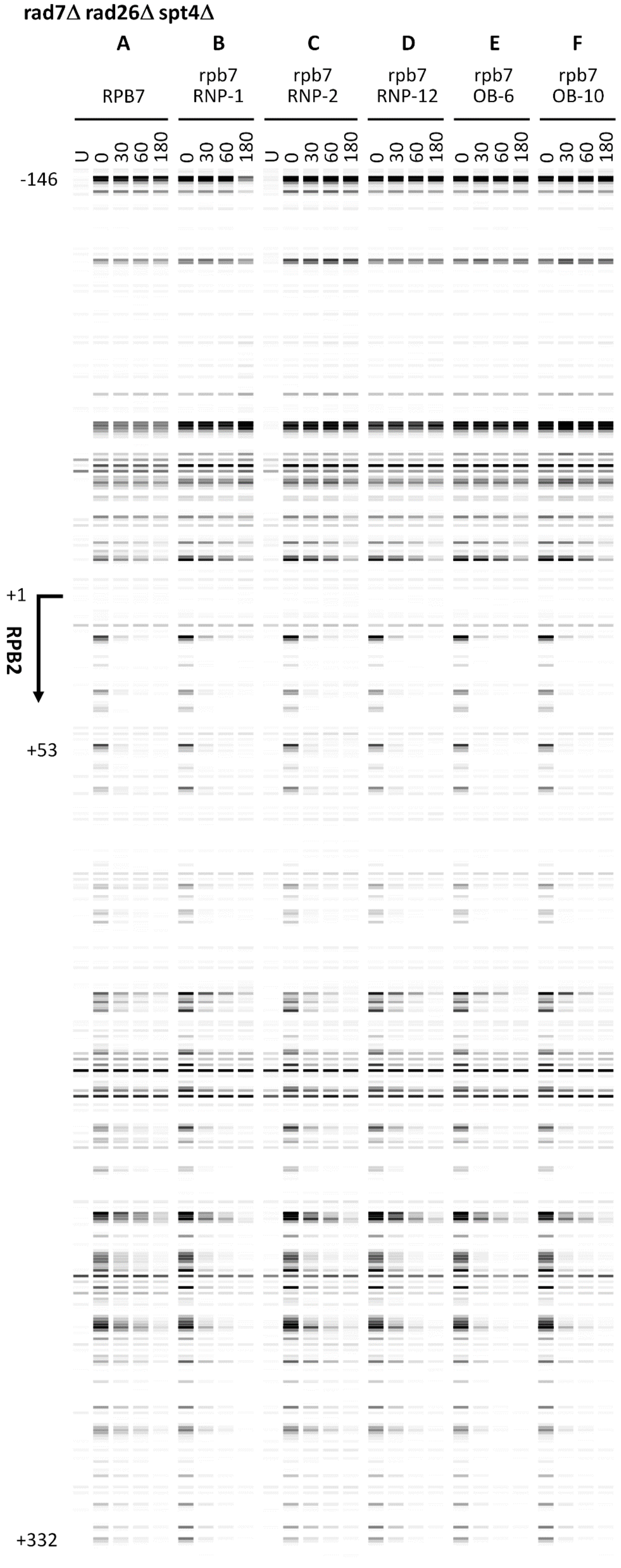


**Supplementary Figure S16.** Pseudo images showing effects of additional Rpb7 mutants on TCR in the *RPB2* gene in *rad7Δ rad26Δ spt4Δ* cells. (A-F) Bands reflecting CPD levels along the TS of *RPB2* genes in *rad7Δ rad26Δ spt4Δ* cells with wild type and mutant Rpb7 at the indicated repair times (min). '*U*', sample unirradiated with UV. Numbers indicating the nucleotide positions of the genes are relative to the major TSS (+1). Note that panel A, which serves as wild type Rpb7 control for comparison with Rpb7 mutants, is the same as Figure 3K.

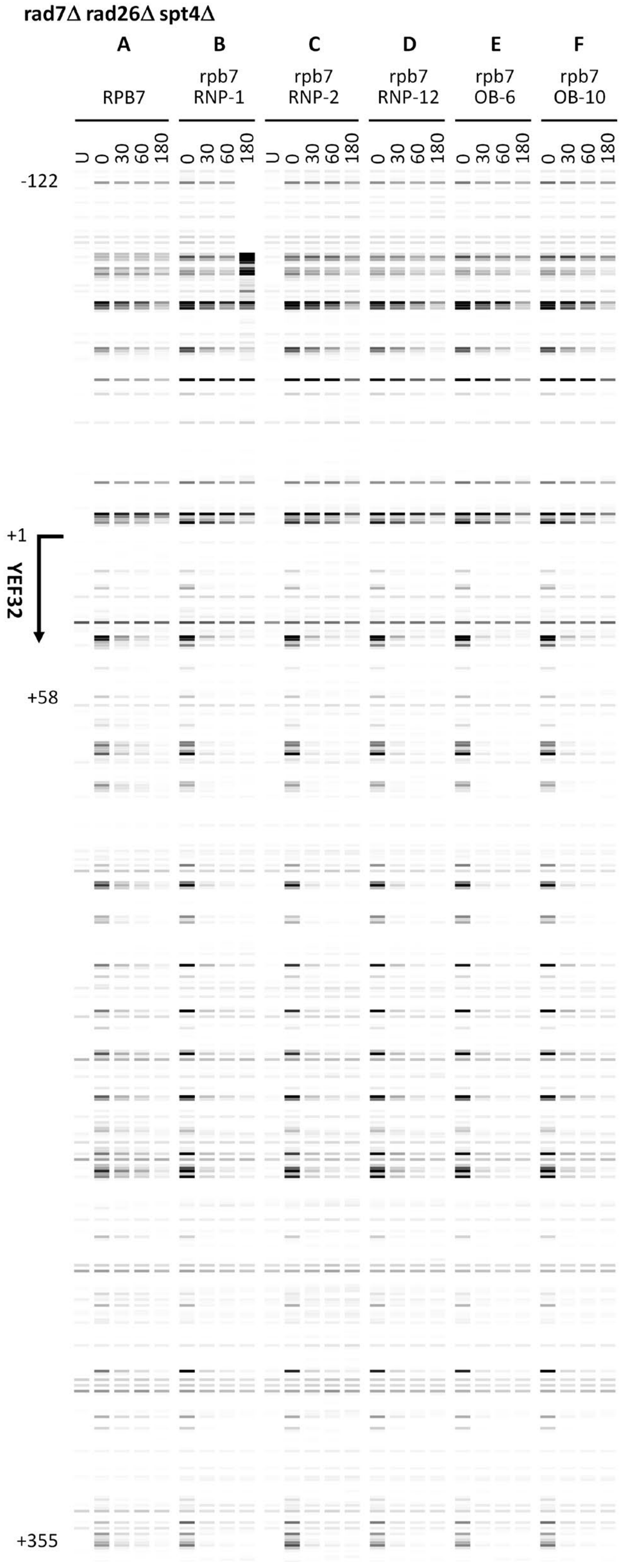


**Supplementary Figure S17.** Pseudo images showing effects of additional Rpb7 mutants on TCR in the *YEF3* gene in *rad7Δ rad26Δ spt4Δ* cells. (A-F) Bands reflecting CPD levels along the TS of *YEF3* genes in *rad7Δ rad26Δ spt4Δ* cells with wild type and mutant Rpb7 at the indicated repair times (min). '*U*', sample unirradiated with UV. Numbers indicating the nucleotide positions of the genes are relative to the major TSS (+1). Note that panel A, which serves as wild type Rpb7 control for comparison with Rpb7 mutants, is the same as Figure 3Q.

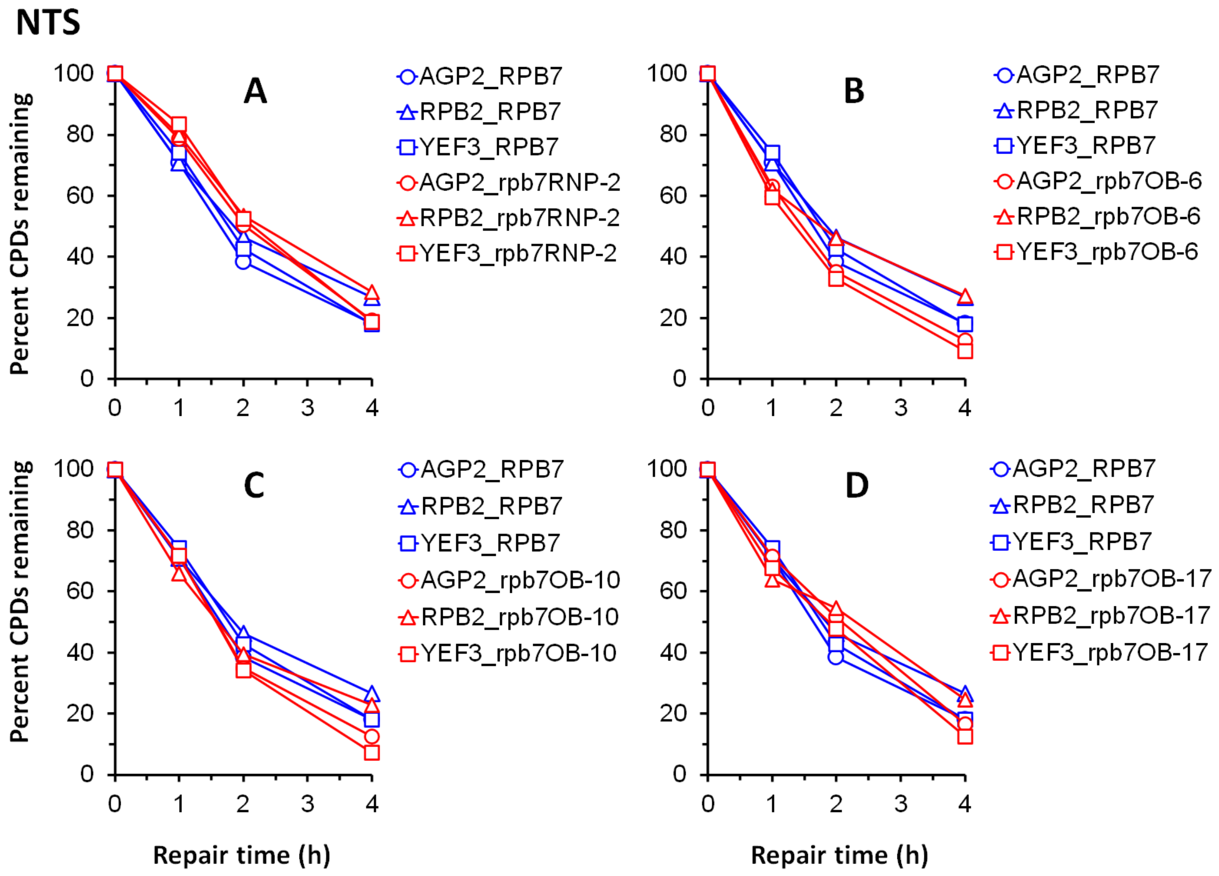


**Supplementary Figure S18.** Plots showing effects of additional Rpb7 mutants on GGR. (A-D) Means of percent CPDs remaining at all CPD sites on the NTS of *AGP2*, *RPB2* and *YEF3* genes in cells with the wild type or indicated mutant Rpb7.

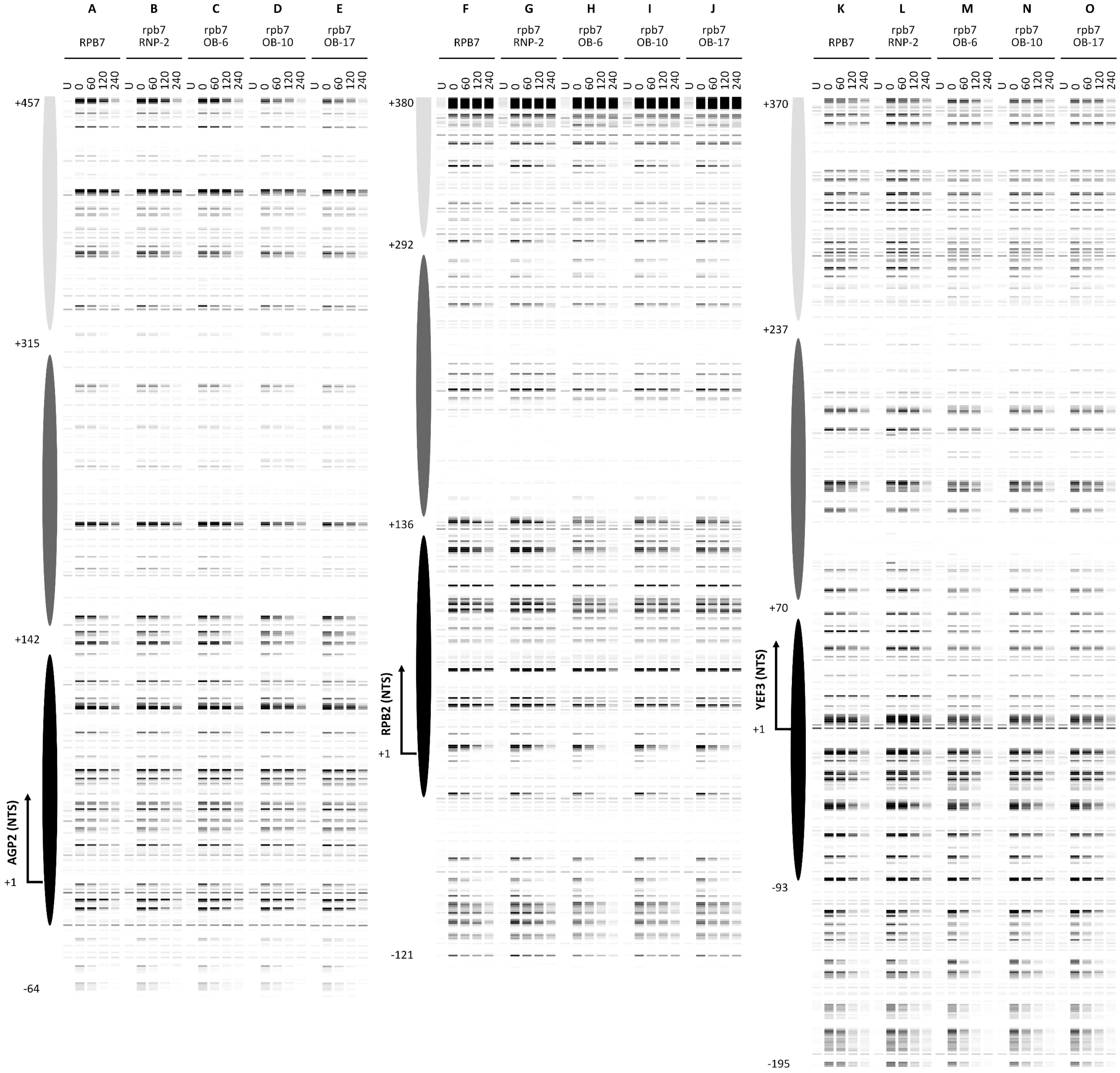


**Supplementary Figure S19.** Pseudo images showing effects of additional Rpb7 mutants on GGR. (A-O) Bands reflecting CPD levels along the NTS of *AGP2*, *RPB2* and *YEF3* genes in cells of indicated genotypes at the indicated repair times (min). '*U*', sample unirradiated with UV. Numbers indicating the nucleotide positions of the genes are relative to the major TSS (+1). Ovals indicate positions of nucleosomes, with darkness reflecting adjacency to the TSS. Note that panels A, F and K, which serve as wild type Rpb7 controls for comparison with Rpb7 mutants, are the same as Figure S1A, C and E, respectively.
